# Effects of mutant lamins on nucleo-cytoskeletal coupling in *Drosophila* models of *LMNA* muscular dystrophy

**DOI:** 10.3389/fcell.2022.934586

**Published:** 2022-08-31

**Authors:** Nicholas M. Shaw, Jose L. Rios-Monterrosa, Gregory R. Fedorchak, Margaret R. Ketterer, Gary S. Coombs, Jan Lammerding, Lori L. Wallrath

**Affiliations:** ^1^ Department of Biochemistry, Carver College of Medicine, University of Iowa, Iowa City, IA, United States; ^2^ The Nancy E. and Peter C. Meinig School of Biomedical Engineering, Weill Institute for Cell and Molecular Biology, Cornell University, Ithaca, NY, United States; ^3^ Biology Department, Waldorf University, Forest City, IA, United States

**Keywords:** myonuclei, muscular dystrophies, *Drosophila*, lamins, LINC complex, microtubules, muscle

## Abstract

The nuclei of multinucleated skeletal muscles experience substantial external force during development and muscle contraction. Protection from such forces is partly provided by lamins, intermediate filaments that form a scaffold lining the inner nuclear membrane. Lamins play a myriad of roles, including maintenance of nuclear shape and stability, mediation of nuclear mechanoresponses, and nucleo-cytoskeletal coupling. Herein, we investigate how disease-causing mutant lamins alter myonuclear properties in response to mechanical force. This was accomplished *via* a novel application of a micropipette harpooning assay applied to larval body wall muscles of *Drosophila* models of lamin-associated muscular dystrophy. The assay enables the measurement of both nuclear deformability and intracellular force transmission between the cytoskeleton and nuclear interior in intact muscle fibers. Our studies revealed that specific mutant lamins increase nuclear deformability while other mutant lamins cause nucleo-cytoskeletal coupling defects, which were associated with loss of microtubular nuclear caging. We found that microtubule caging of the nucleus depended on Msp300, a KASH domain protein that is a component of the linker of nucleoskeleton and cytoskeleton (LINC) complex. Taken together, these findings identified residues in lamins required for connecting the nucleus to the cytoskeleton and suggest that not all muscle disease-causing mutant lamins produce similar defects in subcellular mechanics.

## Introduction

The nuclei of skeletal muscles experience high levels of mechanical force during muscle development and contraction of muscle fibers during work (Rosen & Baylies, 2017; Sweeney & Hammers, 2018). Muscle cells possess structural components to withstand such force, including high expression levels of A-type lamins, lamins A and C (Swift et al., 2013; Cho et al., 2017). Lamins, intermediate filaments that line the inner nuclear membrane, provide structural support for the nucleus, determine the nuclear shape, and organize genomic DNA for proper gene expression (Goldman et al., 2004; Burke & Stewart, 2006; Swift et al., 2013; Zwerger et al., 2013; Camozzi et al., 2014; Dialynas et al., 2015; Kim et al., 2017; Wong et al., 2022). Dominant mutations in the *LMNA* gene encoding the A-type lamins, A and C, cause Emery-Dreifuss muscular dystrophy, limb-girdle muscular dystrophy type 1B, and congenital muscular dystrophy (Bonne et al., 1999; Rankin & Ellard, 2006; Astejada et al., 2007; Worman & Bonne, 2007; Lu et al., 2011; Maggi et al., 2016). Lamins have a conserved domain structure consisting of a head domain, a central coiled-coil rod domain, and a tail domain possessing an Ig-like fold (Dhe-Paganon et al., 2002; Krimm et al., 2002; Mounkes et al., 2003; Herrmann et al., 2007). Lamins dimerize through their rod domain, form filaments through head-to-tail interactions and antiparallel lateral assembly, through lateral contacts that are not well understood to form a meshwork underlying the inner nuclear membrane (Kapinos et al., 2010; Dittmer & Misteli, 2011; Turgay & Medalia, 2017; Eldirany et al., 2021).

The mechanisms by which mutations in the *LMNA* gene cause muscular dystrophy remain incompletely understood and are a topic of intense investigation. Studies in cultured cells and model organisms of muscular dystrophy-associated *LMNA* mutations have revealed insights into potential pathomechanisms (Stewart et al., 2007; Zwerger et al., 2013; Rzepecki & Gruenbaum, 2018; Earle et al., 2020; Coombs et al., 2021). These studies have shown that mutant lamins cause nuclear chromatin protrusions, transient nuclear envelope rupture, increased DNA damage, and abnormal intracellular signaling (Goldman et al., 2004; Dialynas et al., 2015; Hatch & Hetzer, 2016; Earle et al., 2020; Coombs et al., 2021). A potential mechanism to explain these defects is that *LMNA* mutations impair nuclear stability and disrupt nucleo-cytoskeletal connections, which may be particularly devastating in mechanically active tissues such as skeletal and cardiac muscles (Folker et al., 2011; Zwerger et al., 2013; Shin & Worman, 2022).

Cytoskeletal forces are transmitted to the nucleus through the linker of nucleoskeleton and cytoskeleton (LINC) complex (Crisp et al., 2006; Wang et al., 2009; Cain et al., 2018; Hieda, 2019). The LINC complex is comprised of nesprins, proteins that span the outer nuclear membrane and possess a Klarsicht-ANC1-Syne-homology (KASH) domain (Sosa et al., 2012; Wang et al., 2012; Horn, 2014; Kim et al., 2015), and Sad1 and UNC-84 (SUN) domain proteins that span the inner nuclear membrane (Crisp et al., 2006; Wang et al., 2009; Cain et al., 2018; Hieda, 2019). Mammals have six genes encoding a variety of KASH domain proteins, with further complexity generated by alternative splicing (Zhang et al., 2001; Potter & Hodzic, 2018). Mutations in *SYNE1* and *SYNE2*, encoding nesprin 1 and 2, respectively, cause Emery-Dreifuss muscular dystrophy (Zhang et al., 2007; Heller et al., 2020). The nesprin KASH domain connects to the SUN domain proteins across the nuclear lumen. Mammals have two genes encoding SUN proteins, *SUN1* and *SUN2* (Dreger et al., 2001; Hodzic et al., 2004). SUN domain proteins interact with lamins, thereby linking the nucleoskeleton to the cytoskeleton. DNA sequence variation in *SUN1* and *SUN2* might modify muscular dystrophy severity (Meinke et al., 2014).

The goal of this study was to determine the effects of specific mutant lamins on the nuclear shape and nucleo-cytoskeletal coupling in muscle. To achieve this goal, we generated *Drosophila* models of *LMNA* muscular dystrophy possessing either wild-type or mutant *Lamin C* (*LamC*) transgenes. The *LamC* transgenic lines have similar genetic backgrounds, with the exception of the site of insertion of the P-element. *LamC* is an orthologue of human *LMNA* and the only A-type lamin encoded by the *Drosophila* genome. The *Drosophila* LamC protein shares a domain structure that includes 35% amino acid identity and 54% similarity with human lamin A/C. Like human *LMNA*, the expression of endogenous *LamC* is initiated upon cellular differentiation (Riemer et al., 1995). The transcriptional regulators that drive muscle cell differentiation are similar between *Drosophila* and humans (Tapscott et al., 1988; Maire et al., 2020). In both species, differentiated myoblasts fuse to form multinucleated muscle fibers that attach to tendon cells (Ovalle, 1987; Krimm et al., 2002; Schulman et al., 2015; Lemke & Schnorrer, 2018; Richier et al., 2018; Balakrishnan et al., 2021). Thus, much of muscle development and physiology is shared between the two species. Herein, we use the *Drosophila* larval body wall muscles as a proxy for human skeletal muscles. Larval body wall muscles are represented by over 300 individual muscle fibers that can easily be prepared as a muscle fillet (Ramachandran & Budnik, 2010). The use of larval body wall muscles allows for whole organism muscle function assays, cytological analyses, and fillets for *in situ* microharpooning assays to measure nuclear-cytoskeletal coupling and nuclear deformability.

Muscle-specific expression of mutant lamins in an otherwise wild-type *Drosophila* background caused dominant effects on muscle physiology and/or function. These *Drosophila* models of lamin-associated muscle disease revealed vast differences among the mutant lamins tested. Specific mutant lamins altered nuclear shape and succumbed to nuclear envelope deformation under force application. By contrast, other mutant lamins partially uncoupled the nucleus from the cytoskeleton. Interestingly, the domain of lamin affected did not correlate with the loss of a particular nuclear defect, suggesting a complex structure/function relationship, potentially involving additional binding partners. Muscles expressing mutant lamins that caused uncoupling of the nucleus from the cytoskeleton showed a lack of microtubule nuclear caging. A lack of microtubule caging was also observed upon RNAi knockdown of *Msp300*, a *Drosophila* KASH domain protein. Taken together, these data suggest that specific residues within lamin alter nucleo-cytoskeletal coupling that is supported by Msp300. Furthermore, mutation-specific cellular defects suggest that different pathological mechanisms might lead to the common muscle atrophy associated with *LMNA* skeletal muscle disease.

## Materials and methods

### *Drosophila* stocks

*Drosophila* stocks were cultured in cornmeal/sucrose media at 25°C (Shaffer et al., 1994). To generate the *Lamin C* (*LamC*) transgenic lines, full length *LamC* (Gold Clone, accession number AY095046, Berkeley *Drosophila* Genome Project, available from the *Drosophila* Genomics Resource Center, Bloomington, IN) was cloned into the pUAST P-element transformation vector (Brand & Perrimon, 1993). This vector contains a minimal promoter downstream of five upstream activating sequences (UAS) that bind the yeast Gal4 transcriptional activator. Standard embryo injection procedures were used to generate the transgenic stocks in which the P-element was relatively randomly inserted within the genome (BestGene, Chino Hills). Eight to ten independent transgenic lines were established for each *LamC* construct. The site of insertion was mapped to a specific chromosome, and the transgenes were made homozygous. Insertions that were not homozygous viable were discarded. Western analysis was performed, and transgenes that expressed levels of LamC relative to that of the endogenous *LamC* gene were used for analysis. Muscle-specific expression was achieved by crossing the transgenic lines to the *C57* Gal4 driver stock that expresses the yeast Gal4 specifically in larval body wall muscles (Brand and Perrimon, 1993; Gorczyca et al., 2007). The resulting progeny express either wild-type or mutant *LamC* in their larval body wall muscles.

### Viability assays

Genetic crosses were performed in plastic vials with standard medium, cultured at 25°C. Parental flies were removed after larvae were observed in the vials. Dead pupae and live flies were counted for approximately one and a half weeks after the parental adults were removed. Percent viability was calculated by dividing the number of live flies by the total number of live flies plus dead pupae. The total number of living adults plus dead pupae counted for each genotype ranged from 43 to 555. An ANOVA analysis was performed to determine statistical significance. Error bars shown represent 95% confidence interval analyses in GraphPad Prism using the Wilson/Brown method.

### Microharpoon assay and analysis

Microharpoons were generated from borosilicate glass rods (Sutter; OD: 1.0 mm, ID: 0.78, 10 cm length) using a Sutter P-97 micropipette puller. The following parameters were used to achieve tip diameters of ≈1 μm and a suitable taper length: HEAT = 500; PULL = 250; VEL = 220; TIME = 200. *Drosophila* third instar larvae were dissected to expose their body wall muscles. Muscle fillets were secured with magnets on a custom-built, microscope-compatible dissection apparatus and submerged in muscle dissection buffer (128 mM NaCl; 5 mM Hepes, pH 7.4; 2 mM KCl; 35 mM sucrose) supplemented with the fluorescent DNA-binding dye Hoechst 33342 (2 μg/ml) to allow visualization of myonuclei.

The microharpoon assay was performed as previously described (Fedorchak & Lammerding, 2016; Lombardi et al., 2011b), with slight modifications to the pull parameters to accommodate the technique’s use on semi-intact muscle preparations. The microharpoon was inserted into the cytoskeleton ∼10–15 μm from the edge of the nucleus (based on crosshairs in the objective) and pulled 30 μm in the direction away from the nucleus at a rate of 2 μm/s using custom MATLAB software to control the motorized micromanipulator (Eppendorf InjectMan NI2). The pull direction was along the long axis of the myofiber and away from the nucleus. Images were acquired at ×32 magnification (×20 objective with ×1.6 Optivar) every five seconds.

Nucleo-cytoskeletal connectivity was assessed using a custom MATLAB program (MATLAB 2010, Natick, MA) available upon request. For each nucleus, the user first selected a binary threshold value from a histogram of “pixel count versus intensity” to account for heterogeneity in the Hoechst 33342 signal, which the program thresholds to provide an accurate trace of the nucleus during deformation. After applying erosion and dilation processing steps to smooth the thresholded nucleus, the program uses the MATLAB “regionprops” function to track the centroid of the nucleus, generate a nuclear bounding box, and extract the necessary parameters to fit an ellipse to the nucleus. Changes in nuclear strain, centroid displacement, and additional parameters were computed. For most calculations, the frame prior to micropipette harpoon removal (frame of maximum pull) was compared with the first frame before the initiation of the pull. Myonuclei were excluded from analysis if 1) the myofiber was at an angle > ±30° from the vertical axis, 2) the micropipette harpoon was not inserted at the proper distance (≈10–15 µm) from the nuclear envelope, 3) the microharpoon failed to enter the cytoplasm and instead brushed over the top of the fiber, and 4) the myofiber retracted during or after the micropipette harpoon pull.

### Immunohistochemistry

Third instar larval body wall muscle dissections were performed according to published procedures (Budnik et al., 1990). After fixation in 4% paraformaldehyde, preparations were stored in 1X PBS. Muscle fillets were washed three times in 1X PBS for five minutes each wash. Muscle fillets were then washed three times in permeabilization buffer (1X PBS, 0.5% Triton-X-100, and 5 mM MgCl_2_) for five minutes each wash. Preparations were stained with Texas Red^®^-X Phalloidin (1:400 dilution) in permeabilization buffer containing 0.5% boiled/filtered fish skin gelatin (G-7765, Sigma-Aldrich, St. Louis) and stained with either guinea pig anti-Msp300 antibodies (kind gift from T. Volk; 1:100 dilution), mouse anti-Klar-C (#9C10, Developmental Studies Hybridoma Bank University of Iowa, Iowa City 1:25 dilution), rat anti-Koi (kind gift from J.A. Fischer; either 1:20 or 1:50 dilution), mouse anti-α-tubulin (#12G10, Developmental Studies Hybridoma Bank University of Iowa, Iowa City; 1:200 dilution), and mouse anti-lamin C (1:200 dilution) or mouse anti-lamin Dm_0_ (#ADL84.12; Developmental Studies Hybridoma Bank University of Iowa, Iowa City; 1:400 dilution). Nuclear pores were stained with MAb414 (ab24609, Abcam, Cambridge, United Kingdom; mouse monoclonal antibody, 1:2,000 dilution). Microscopy was performed using either a Leica DMLB phase contrast fluorescence microscope and a Zeiss 710 confocal microscope or a Leica Thunder fluorescence microscope. Three channel images were merged to produce composite images using ImageJ.

Quantification of the cytoplasmic and nuclear immunofluorescent signal was performed using the integration intensity feature of Fiji (Schindelin et al., 2012). Briefly, nuclei were selected as regions of interest (ROI) using the cell counting function. Muscle fibers were visualized by phalloidin staining and outlined using the trace tool. Nuclear staining was quantified using the integrative intensity feature. A cytoplasmic signal was quantified by subtracting the integrative intensity of the nuclei from the total signal intensity. The correction factor was calculated by drawing a circle in an area lacking muscle and measuring the integrated intensity. This measurement was divided by the area of the circle to yield the correction factor. The background signal was determined by multiplying the area of the ROI by the correction factor, then subtracting this value from the raw integrated intensity values.

Quantification of perinuclear microtubular staining was performed visually by three individuals blinded to the genotype of the larvae. The final number of nuclei assigned to each phenotype represents the average of the three measurements. Each phenotype is represented as a percent of the total nucleus score.

### Larval motility assays

Quantitative measurements of larval velocity (mm/min) were made for the host stock (*w* ^*1118*^) and larvae expressing either wild-type or mutant *LamC* in larval body wall muscles *via* the *C57* Gal4 driver (Gorczyca, 2007; Brand, 1993). Motility was also quantified for larvae with body wall muscle-specific expression of either *luciferase* RNAi or an RNAi transgene against a LINC complex component. For motility assays, third instar larvae were placed on a room temperature 1.8% agarose-filled 15-cm Petri dish for ten minutes to allow adjustment to a new substrate. Following, five larvae were placed in the center of a second 1.8% agarose-filled Petri dish marked with concentric circles. Their crawling was videotaped for 2 minutes using a cell phone. Also, the videos were analyzed using wrMTack, a plug-in for ImageJ (Brooks et al., 2016). A calibration line was drawn from the first concentric circle to the second using the “line tool” to produce the calibration length value. The path crawled by each larva was traced by generating a Z-project using the max intensity function in ImageJ. Distances traveled were measured by tracing larval paths with the segmented line drawing tool. Distances were divided by the calibration length value, resulting in the distance in millimeters. Velocities were calculated by dividing the distance traveled by the recording time (120 s). Two groups of five larvae were measured per genotype. The resulting values were plotted using GraphPad Prism (GraphPad Prism version 8.0.0 for Mac, GraphPad Software, San Diego, California, United States), and velocities were compared to those of the wild type using a one-way ANOVA with Holm-Sidak’s correction.

### Statistics

Groups of datasets were analyzed using a one-way analysis of variance (ANOVA). Comparisons between two datasets that showed a normal distribution were analyzed using the Student’s *t*-test. Excel (16051.14931.20132.0, Microsoft, Seattle) and GraphPad Prism (v.8.0.0 for Mac, GraphPad Software, San Diego) were used to generate graphs and perform statistical analyses. Error bars in graphs represent means ± standard deviation (SD) unless otherwise noted. All results reported were derived from a minimum of three independent biological samples.

## Results

### *Drosophila* larval body wall muscle-specific expression of mutant lamins caused premature death

**FIGURE 1:**
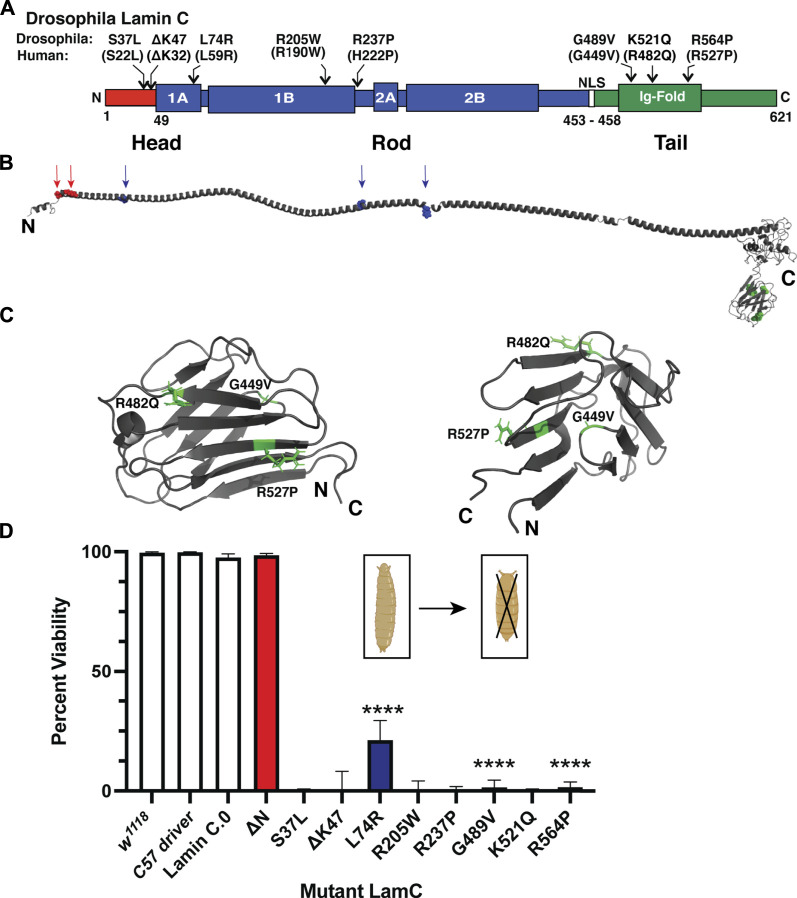
Muscle-specific expression of mutant *LamC* causes premature death. **(A)** Diagram of the *Drosophila* LamC domain structure with the head (red), rod (blue), and tail (green) domains indicated. The positions of the amino acid changes examined in this study are shown with the corresponding human amino acid changes in parentheses. **(B)** Three-dimensional model of full-length human lamin A (Hinz et al., 2021) with the position of the amino acid changes in the head and rod domain indicated by arrows. The positions of amino acid changes in the Ig fold are indicated in the magnified view (panel C). **(C)** Enlarged views of the three-dimensional lamin A/C Ig-fold domain based on an NMR structure (PBD 1IVT) (Krimm et al., 2002) with the amino acid substitutions examined here labeled. **(D)** Larvae with body wall muscle-specific expression of wild-type and mutant *LamC* were allowed to develop and scored for adult viability. Percent viability was calculated by dividing the number of live adults by the total number of offspring (adult flies plus dead pupae). The X-axis shows the genotype, and the Y-axis shows the percent viability. Larvae expressing *LamC* S37L, ΔK47, R205W, R237P, and K521Q yielded no surviving adults. Larvae expressing *LamC* L74R, G489V, and R564P had a significant reduction in survivability to adulthood. Colors correspond to the protein domain where the amino acid change is located: head (red), rod (blue), and tail (green). Fisher’s exact test was used to compare values for the mutants versus the wild-type control. Total progeny scored per genotype ranged from 43 to 555. Error bars represent 95% confidence intervals. ****, *p* ≤ 0.0001. Bio-icons in panel D were created with BioRender.com.

We modeled eight *LMNA* mutations in *Drosophila LamC* that were selected based on their causality in human disease (Figure 1A; Table 1). These mutations altered amino acids in all three lamin protein domains. Some of the amino acid changes affected amino acids that are identical between human lamin A/C and *Drosophila* LamC (S37L, K47, L74, R205, and R237), while others are represented by conservative substitutions (R237 and K521).

**TABLE 1:** Lamin A/C amino acid changes examined in this study.

Human a.a. change	Drosophila a.a. change	Domain	Muscular phenotype	Reference
ΔN	ΔN	Head	AD-EDMD	Walter et al. (2005)
S22L	S37L	Head	DCM	Pethig et al. (2005)
ΔK32	ΔK47	Head	AD-EDMD and dropped head syndrome	Muchir et al. (2004) and D'Amico et al. (2005)
L59R	K74R	Rod	DCM	McPherson (2009)
R190W	R205W	Rod	DCM	Pethig et al. (2005), Arbustini et al. (2002), Hermida-Prieto et al. (2004), Sylvius et al. (2005), and Song et al. (2007)
H222P	R237P	Rod	EDMD	Bonne et al. (2000)
G449V	G489V	Tail	Striated muscle laminopathy	Dialynas et al. (2010)
R482Q	K521Q	Tail	AR-EDMD	Wiltshire et al. (2013)
R527P	R564P	Tail	AD-EDMD	Bonne et al. (1999) and Brown et al. (2001)

The amino acid changes under investigation were mapped onto the three-dimensional structure of lamin A/C that is based on a combination of experimental data and *in silico* predictions (Figure 1B) (Krimm et al., 2002; Strelkov et al., 2004; Lilina et al., 2020; Hinz et al., 2021). The amino acid residues S22 and K32 map within a short-predicted alpha-helical region of the head domain (Figure 1B). The amino acid residues L59, R190, and H22 map within the long alpha-helical regions of the rod domain (“coiled coil”). The amino acid residue G449V maps to a loop region within the Ig fold (Figure 1C). By contrast, amino acid residues R482 and R527 map to β-sheets in the Ig fold (Figure 1C). Thus, by testing this set of amino acid changes, we are surveying all three conserved lamin domains.

To determine the effects of these mutant lamins on the physical properties of myonuclei, we expressed the mutant lamins in *Drosophila* larval body wall muscles using the tissue-specific Gal4/UAS system (Caygill & Brand, 2016). The *C57* Gal4 driver stock expresses the Gal4 transcription factor in larval body wall muscles throughout larval development (Brand & Perrimon, 1993; Gorczyca et al., 2007). These flies were crossed with flies expressing either wild-type or mutant *LamC* transgenes. Larvae with body wall muscle-specific expression of wild-type *LamC* developed normally; they crawled up the vial wall, underwent metamorphosis to pupae, and emerged as adults with no apparent defects. By contrast, larvae with body wall-specific expression of mutant *LamC* exhibited difficulties crawling up the vial wall and died during the late pupal stage. Death at this stage is consistent with loss of larval body wall muscle function, which is required for morphogenesis (Fortier et al., 2003; Dialynas et al., 2010). To quantify the effects of the mutant lamins on viability, the fraction of viable adults was calculated by dividing the number of live adult flies by the number of total progeny (live plus dead pupae). A small percentage of viable adults resulted from the expression of LamC L74R, G489V, and R564P (Figure 1D). However, no adult progeny resulted upon expression of LamC S37L, ΔK47, R205W, R237P, and K521Q (Figure 1D). Western analysis of protein extracted from larval carcasses (mostly body wall muscle) revealed that the lethality was caused by the mutant LamC, not by general over-expression of LamC. LamC levels were similar in all genotypes, except for R564P, which had high variable expression among biological replicates (Supplementary Figures S1A, B). Thus, amino acid substitutions in all three lamin domains cause lethality at the pupal stage.

### Specific mutant lamins cause nuclear envelope protein mislocalization

**FIGURE 2:**
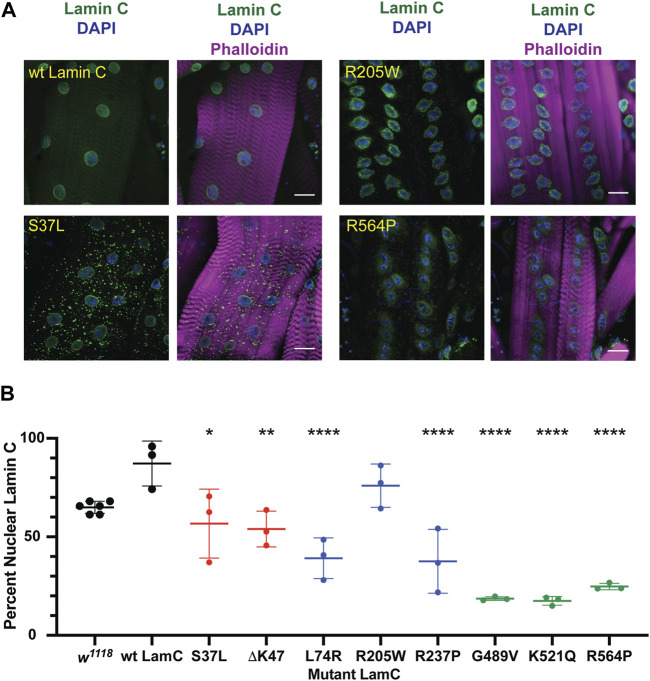
Muscle-specific expression of mutant *LamC* causes cytoplasmic lamin aggregation. (**A**) Third instar larval body wall muscles expressing either wild-type or mutant LamC were dissected and stained with antibodies to LamC (green), phalloidin (magenta), and DAPI (blue). Representative images for an amino acid substitution in each of the three LamC domains are shown. Images of muscle expressing all of the amino acid changes examined here are shown in Supplementary Figure S2. Scale bar: 30 μm. (**B**) Percentage of nuclear LamC was quantified by measuring the intensity of nuclear LamC antibody staining divided by the total amount of staining in the muscle cell (i.e., nuclear plus cytoplasmic LamC) and multiplying by 100. Three to six muscle fibers containing five to 29 nuclei from two to three larvae were analyzed. Each data point represents the average percent nuclear signal in a muscle fiber. The mean and standard deviation of the values obtained for muscle fibers of each genotype are shown. A one-way ANOVA analysis was used to determine statistical significance. *, *p* ≤ 0.05; **, *p* ≤ 0.01; ***, ≤0.001; ****, *p* ≤ 0.0001.

To investigate the cause of lethality due to mutant versions of LamC at the cellular level, immunohistochemistry was performed on larval body wall muscles. Muscles expressing either wild-type or mutant *LamC* transgenes were stained with antibodies against LamC, lamin Dm_0_ (the only B-type lamin in *Drosophila*), and FG-repeat containing nuclear pore proteins. Antibody staining showed that wild-type LamC localization was confined to the nucleus, particularly the nuclear envelope, as observed for the host stock *w* ^*1118*^ (Figure 2; Supplementary Figure S2). Myonuclei expressing LamC R205W were abnormally shaped, with LamC confined to the nucleus; however, nuclear aggregates were also apparent (Figure 2; Supplementary Figure S2). By contrast, myonuclei expressing LamC S37L, L74R, G489V, K521Q, and R564P had a spherical nuclear shape and showed cytoplasmic aggregation of LamC. Thus, the mutant lamins exhibited a range of abnormal cellular localization patterns that did not correlate with the domain of lamin affected.

In humans, A- and B-type lamins form independent networks underlying the inner nuclear envelope (Shimi et al., 2015). To determine if the mutant LamC proteins perturbed the distribution of the *Drosophila* B-type lamin, lamin Dm_0_, larval body wall muscles expressing either wild-type or mutant *LamC* transgenes were stained with antibodies specific to lamin Dm_0_. Neither expression of wild-type LamC nor mutant LamC perturbed the localization of lamin Dm_0_ at the nuclear envelope (Supplementary Figure S3). These data strongly suggest that the two lamin types form independent networks in *Drosophila*, like in humans, and that mutant LamC does not overtly interfere with lamin Dm_0_ organization.

**FIGURE 3:**
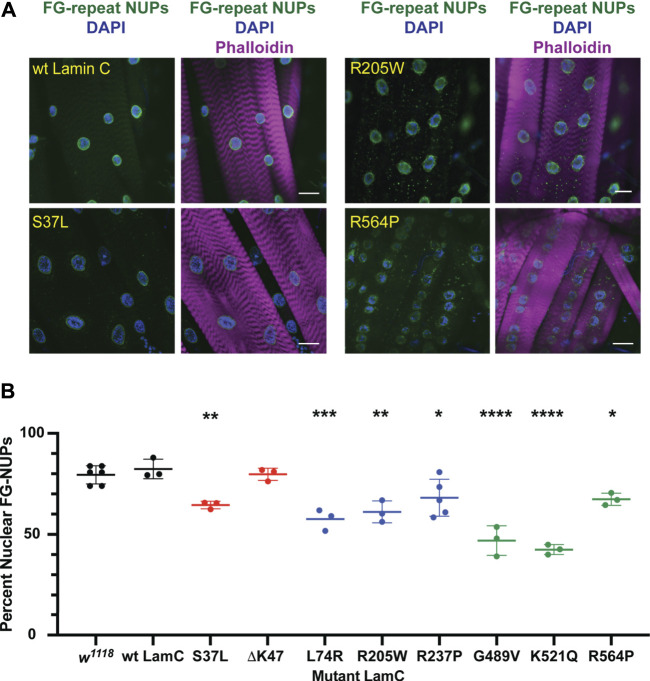
Mutant *LamC* alters the localization of FG-containing nuclear pore proteins. **(A)** Third instar larval body wall muscles expressing wild-type or mutant LamC were dissected and stained with antibodies to FG-repeat-containing nuclear pore proteins (NUPs) (green), phalloidin (magenta), and DAPI (blue). Representative images for an amino acid substitution in each of the three LamC domains are shown. Results from all amino acid changes studied in this manuscript are shown in Supplementary Figure S4. Scale bar: 30 μm. **(B)** Percent of nuclear NUP staining was quantified by measuring the intensity of the nuclear FG-repeat antibody staining divided by the total amount of staining (nuclear plus cytoplasmic), then multiplying by 100. Three to six muscle fibers containing three to 28 nuclei from two to three larvae were analyzed. Each data point represents the average percent nuclear signal in a muscle fiber. The mean and standard deviation of the values obtained for muscle fibers of each genotype are shown. A one-way ANOVA analysis was used to determine statistical significance. *, *p* ≤ 0.05; **, *p* ≤ 0.01; ***, ≤0.001; ****, *p* ≤ 0.0001.

Lamins interact with many different proteins in the nucleus, including those that make up nuclear pores (NUPs) (Wilson & Foisner, 2010; Kittisopikul et al., 2021). To determine if the mutant lamins altered the localization of nuclear pore proteins, larval body wall muscles expressing either wild-type or mutant *LamC* transgenes were stained with antibodies to FG-repeat-containing NUPs. As anticipated, muscles expressing wild-type *LamC* showed punctate anti-NUP staining at the nuclear envelope (Figure 3; Supplementary Figure S4). In contrast, in muscles expressing LamC ΔK47, the anti-NUP staining was distributed unevenly around the nuclear envelope (Supplementary Figure S4). Perhaps this reflects the involvement of lamins in the proper spacing of nuclear pores (Furukawa et al., 2009; Kittisopikul et al., 2021). Intriguingly, in muscles expressing LamC R205W, R237P, G489V, K521Q, and R564P, the NUPs mislocalized to the cytoplasm (Figure 3; Supplementary Figure S4). Mislocalization may cause depletion of the NUPs in the envelope, affecting nuclear–cytoplasmic transport. In addition, cytoplasmic aggregation of NUPs could be toxic to cytoplasmic events such as intracellular signaling and maintenance of redox homeostasis (Rajasekaran et al., 2007; Chivet et al., 2020; Bobylev et al., 2021; Coombs et al., 2021; Potulska-Chromik et al., 2021). Thus, the mutant lamins had differing effects on NUP localization with several residues in the rod and Ig-like fold domains implicated in proper NUP formation. Substitution at these residues caused NUP cytoplasmic aggregation, suggesting the formation of an abnormal A-type lamin meshwork does not support proper assembly or maintenance of the NUPs.

### Specific mutant lamins cause nucleo-cytoskeletal uncoupling and nuclear deformation

To understand the functional consequences of the mutant lamins on the mechanical properties of myonuclei, we employed a novel application of a microharpooning assay. This assay allows for quantitative assessment of nucleo-cytoskeletal coupling and nuclear deformation under local force application to the perinuclear cytoskeleton (Maniotis et al., 1997; Lombardi et al., 2011a; Fedorchak & Lammerding, 2016). The assay uses a fine-tipped glass needle to “harpoon” the cytoskeleton at a defined position near the nucleus and then applies a precisely defined displacement while monitoring induced nuclear displacement and deformation *via* real-time fluorescence microscopy imaging. The application described here was the first use of this technique on living, multinucleated muscle fibers *in situ*. For standard preparation of the larval body wall muscle, a longitudinal cut is made at the ventral midline of the larvae, organs are removed, and the remaining tissue, termed a muscle fillet, represents body wall muscles, attached to tendon cells adhered to the hypodermis (Carayon et al., 2020).

**FIGURE 4:**
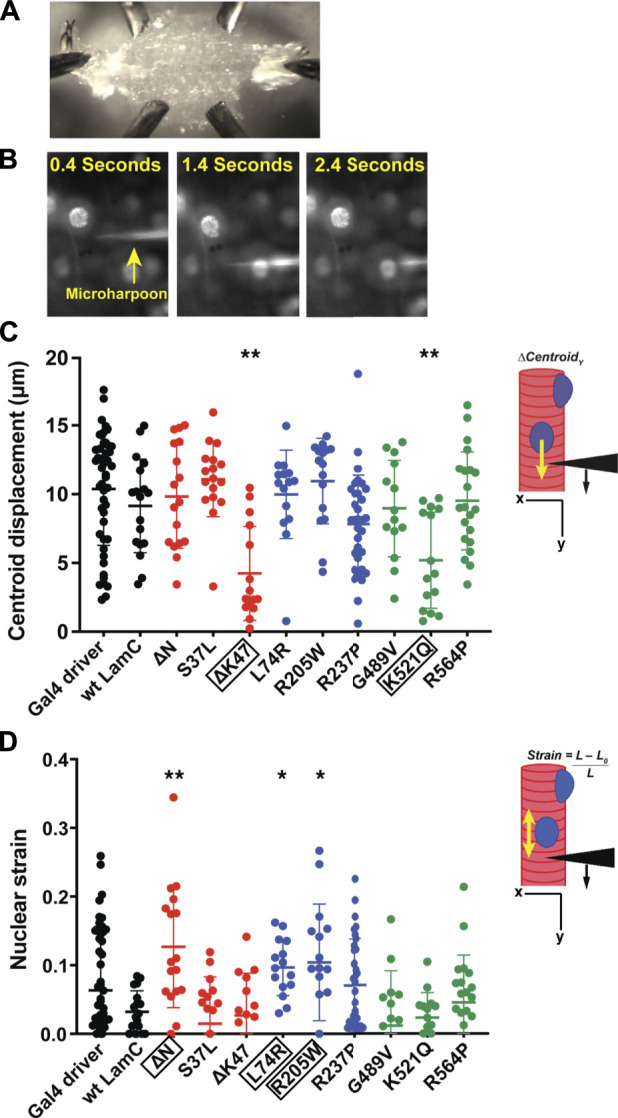
Specific mutant lamins reduce nucleoskeletal-cytoskeletal coupling and increase nuclear deformation. Body wall muscles from larvae expressing either wild-type or mutant LamC were dissected and maintained in a physiological buffer. **(A)** Representative semi-intact larval body wall muscle fillet is shown. Such muscle fillets were used for micropipette harpooning assays to measure physical coupling between the nucleus and cytoplasm and nuclear strain. **(B)** Representative time-lapse images from a video of microharpooning wild-type muscles. Images shown were taken at 0.4, 1.4, and 2.4 s from the video recording. **(C)** Inset diagram shows the experimental approach with the black arrowhead representing a micropipette, the black arrow indicating the direction of force application, and the yellow arrow representing the movement of the center point of the nucleus, centroid displacement, which is a change in position in the y-direction. Expression of wild-type LamC did not alter centroid displacement compared to that of the Gal4 driver control. In contrast, centroid displacement was reduced for larvae expressing LamC ΔK47 and K521Q, indicating a loss of nucleoskeleton-cytoskeleton coupling. Colors correspond to the domains in which each amino acid change is located: head (red), rod (blue), and tail (green). An ANOVA analysis was used to determine statistical significance among the genotypes. Boxed amino acids are those with statistical differences; 15–48 nuclei were analyzed per genotype. *, *p* ≤ 0.05; **; *p* ≤ 0.01. **(D)** Inset diagram shows the experimental approach with the black arrowhead representing a micropipette needle, the black arrow indicating the direction of force application, and the yellow double-headed arrow representing the measured change in nuclear deformation. Nuclear strain is measured as the length of the nucleus at the end of the force application minus the length of the nucleus upon initial force application divided by the initial length of the nucleus. Expression of wild-type LamC did not alter nuclear strain compared to that of the Gal4 driver-only control. In contrast, muscles expressing LamC ΔN, L74R, and R205W showed increased nuclear strain compared to the controls. An ANOVA analysis was used to determine statistical significance. Boxed amino acids are those with statistical differences; 15–48 nuclei were analyzed per genotype. *, *p* ≤ 0.05; **, *p* ≤ 0.01.

For our studies, larval body wall muscles were dissected, immobilized on a glass slide (Figure 4A), and harpooned in a physiological buffer while mounted on a microscope. A fine-tipped glass needle attached to a computer-controlled micromanipulator was inserted 10–15 μm from the edge of a nucleus and pulled 30 μm at a rate of 2 μm/s. The direction of pull was along the long axis of the muscle fiber and away from the nucleus. Images were captured by time-lapse microscopy for five seconds (Figure 4B; Supplementary Videos S1–S4). From the recorded videos, two types of subcellular mechanical measurements were taken. First, the distance the center of the nucleus was displaced from its original position upon force application by the microharpoon (“nuclear centroid displacement”) served as a quantitative measurement of nucleo-cytoskeletal coupling (Figure 4C). Defects in nucleo-cytoskeletal coupling are expected to result in reduced nuclear centroid displacement (Lombardi et al., 2011b). Second, the extent of nuclear elongation upon force application, normalized to the initial length of the nucleus (“nuclear strain”), served as a quantitative measurement of nuclear deformability (Figure 4D). Relative to the controls, only muscles expressing LamC ΔK47 and K521Q showed a significant decrease in nuclear centroid displacement (Figure 4C), suggesting that these mutant lamins partially uncoupled the nucleus from the cytoskeleton. Compared to controls, only LamC ΔN, L74R, and R205W showed significantly increased nuclear deformation (Figure 4D), suggesting reduced nuclear mechanical stability. We recognize that nuclear deformation in the microharpoon assay can also be influenced by the transmitted force, so an increase in nuclear deformation could also result from increased force transmission to the nucleus, but the more likely explanation appears to be an increase in nuclear deformability. Surprisingly, some mutant lamins (S37L, R237P, G489V, and R564P) had no apparent effect on either nuclear-cytoskeletal coupling or nuclear deformability (Figure 4D). Such findings suggest that these mutants might cause defects unrelated to the mechanical properties of the nucleus such as genome organization. Collectively, these data show that mutant lamins have distinct effects on the physical properties of the nucleus regardless of the lamin domain affected.

### Mutant lamins that reduce nucleo-cytoskeletal coupling exhibit loss of myonuclear microtubule caging

**FIGURE 5:**
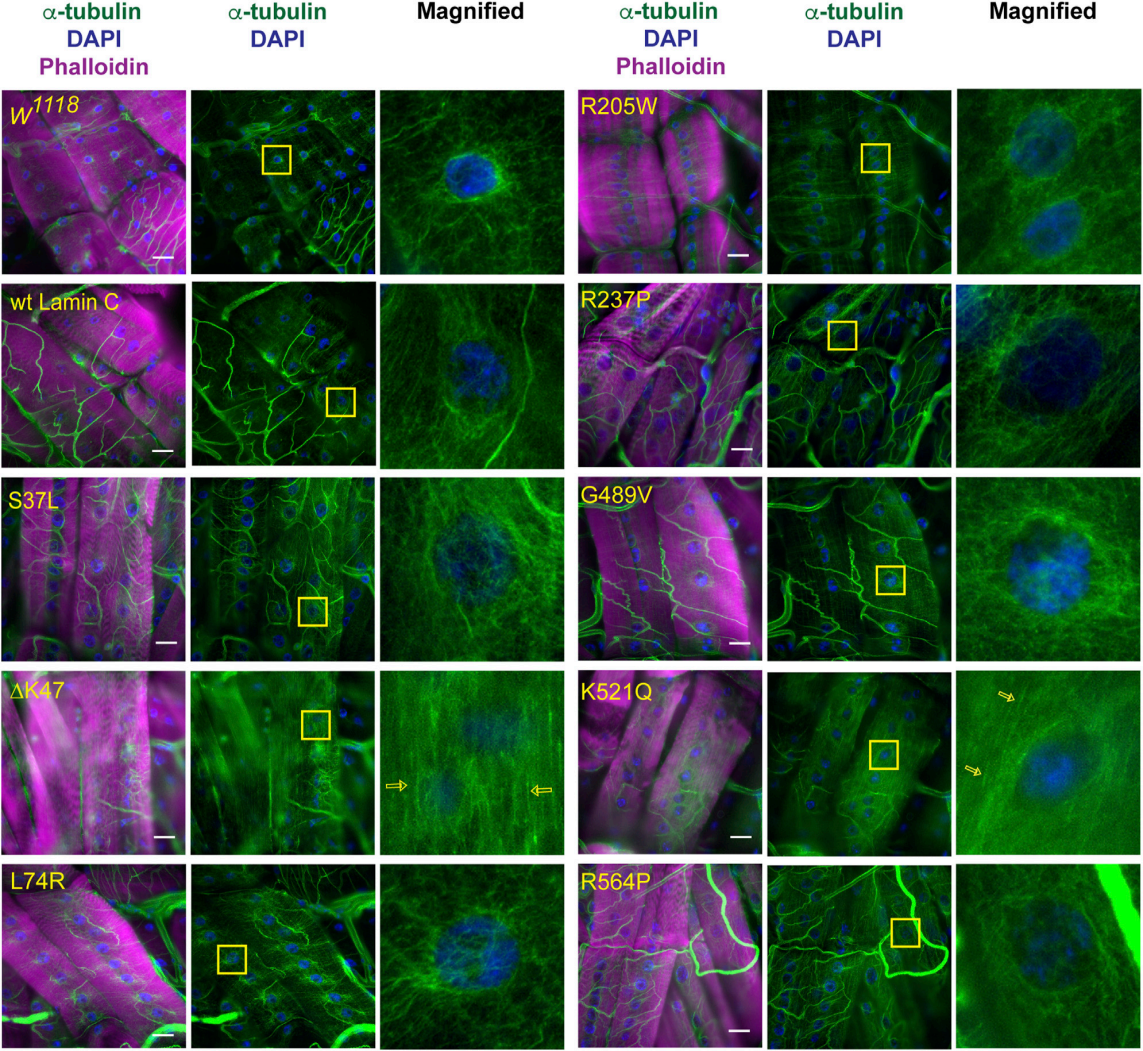
Specific mutant lamins cause loss of microtubular caging around the nucleus. Third instar larval body wall muscles expressing either wild-type or mutant LamC were stained with antibodies to α-tubulin (green and white in magnified images), phalloidin (magenta), and DAPI (blue). In wild-type muscle, α-tubulin forms a cage around the nucleus as observed for the host stock *w* ^*1118*^ and muscles expressing wild-type LamC. Expression of the majority of mutant lamins did not alter the α-tubulin nuclear cage. However, the cage was not apparent in muscles expressing mutant LamC ΔK47 and K521Q. In these cases, the microtubules were arrayed parallel to the length of the muscle fiber (open yellow arrows). Yellow boxes indicate the area magnified in the right panels. Scale Bar: 30 μm.

Given the lack of an apparent correlation between nuclear mechanical defects and nuclear envelope protein localization, we examined perinuclear cytoskeletal organization as a potential factor. Based on the muscle staining of phalloidin, which binds actin (Supplementary Figures S2–S4), and anti-alpha-actinin, which crosslinks actin (Supplementary Figure S5), no overt muscle-wide defects in actin organization were apparent in the muscles expressing mutant LamC at the light microscope level; however, they could be present. Other typical components of the cytoskeleton include cytoplasmic intermediate filaments and microtubules. However, the *Drosophila* genome does not contain genes encoding the standard cytoplasmic intermediate filaments such as desmin and vimentin (Herrmann & Strelkov, 2011; Cho et al., 2016). Therefore, we stained with antibodies to α-tubulin, which revealed striking differences among the mutant lamins in perinuclear microtubule organization. In larval body wall muscles expressing wild-type *LamC*, microtubules form a cage around the nucleus like that observed for the host stock (Figure 5). Similar microtubule cages have also been observed in mammalian cells (Bruusgaard et al., 2006; Earle et al., 2020). The α-tubulin staining pattern of muscles expressing LamC S37L, L74R, R205W, R237P, G489V, and R564P gave similar results to that of the wild type (Figure 5). By contrast, α-tubulin staining of muscles expressing LamC ΔK47 and K521Q showed a loss of microtubule caging around the nucleus. Instead, the microtubules were aligned orthogonal to the long axis of the muscle fiber and did not show any enrichment at the nucleus (Figure 5). Interestingly, the lack of myonuclear microtubule caging was only observed for the two mutants that eliminated nucleo-cytoskeletal coupling and not in the other mutants that had normal nucleo-cytoskeletal force transmission. This suggests that microtubules are critical components for the nucleo-cytoskeletal coupling in *Drosophila* larval body wall muscles. This idea is supported by the fact that the mutant versions of LamC that cause loss of nuclear microtubule caging also exhibit larval motility defects (Supplementary Figure S6).

### RNAi knockdown of the LINC complex component Msp300 recapitulates the loss of nuclear microtubule caging

**FIGURE 6:**
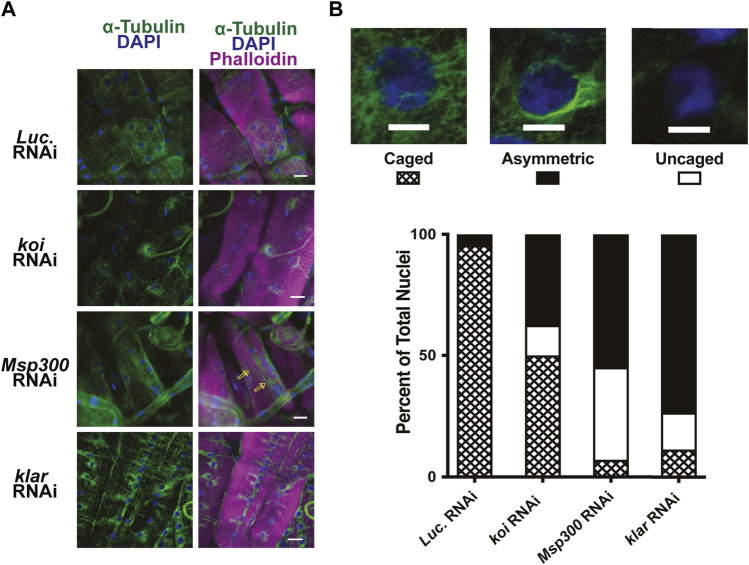
RNAi against LINC complex components disrupts perinuclear microtubule organization. **(A)** Immunohistochemistry was performed on third instar larval body wall muscles expressing either RNAi against *Luciferase* (control) or LINC complex components using antibodies to α-tubulin (green), phalloidin (magenta), and DAPI (blue). Open yellow arrows indicate microtubules that run parallel with the long axis of the muscle fiber, giving rise to uncaged nuclei. **(B)** Representative patterns of microtubule organization around the nucleus are shown at the top. Scale Bar: 10 mm. The graph represents the percentage of nuclei showing each pattern of localization per genotype. Muscles expressing an RNAi against *Luciferase* (*luc*) show nearly 100% caged nuclei. In contrast, RNAi knockdown of each of the LINC complex members shows increased numbers of asymmetric and uncaged nuclei. A range of 32–79 nuclei were scored per genotype. Scale Bar: 30 μm.

Nesprins are components of the LINC complex and have been shown in some cases to interact with microtubules (Chang et al., 2015; Starr, 2017; Maurer & Lammerding, 2019). The *Drosophila* LINC complex consists of a single SUN domain protein Klaroid (Koi), encoding the SUN domain protein, and either Msp300 or Klarischt (Klar) as the KASH domain proteins (Yu et al., 2006; Kracklauer et al., 2007; Technau & Roth, 2008; Xie & Fischer, 2008). To assay for the involvement of the LINC complex in perinuclear microtubule organization in *Drosophila* larval body wall muscles, we used RNAi transgenes to deplete each of the LINC complex components separately in larval body wall muscles. Effective depletion of each protein was confirmed by immunofluorescence, with RNAi targeting *luciferase* as a negative control (Supplementary Figure S7). Larval body wall muscles typically show nuclear microtubule caging (Figure 5). In muscles expressing the *luciferase* RNAi control, more than 90% of the myonuclei had the expected microtubule caging (Figures 6A, B). By contrast, larval body wall muscles with depletion of LINC complex components showed a range of myonuclear phenotypes (Figure 6B). Some myonuclei displayed an asymmetrical arrangement of microtubules, with enrichment of microtubules on one side of the nucleus. Other myonuclei completely lacked microtubule caging. Msp300 depletion produced the greatest number of myonuclei that lacked microtubule caging compared to the other genotypes. In the Msp300 depleted cells, the microtubules were aligned orthogonal to the long axis of the muscle fiber, remarkably like the microtubule arrangement in muscles expressing LamC ΔK47 and K521Q (Figure 5). These findings suggest that Msp300 is a crucial link between lamins and perinuclear microtubules.

**FIGURE 7:**
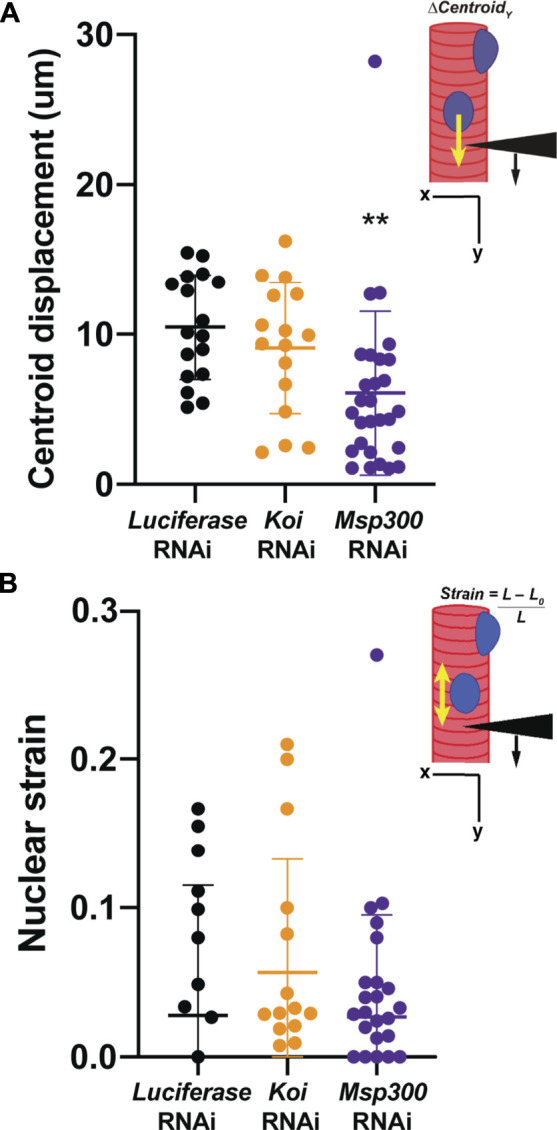
Muscle-specific RNAi against *Msp300* causes loss of nucleoskeletal-cytoskeletal coupling. The micropipette harpooning assay was performed on larval body wall muscles expressing an RNAi transgene against either *Luciferase* (as a control) or a LINC complex component. **(A)** Inset diagram shows the experimental approach with the black arrowhead representing a micropipette, the black arrow indicating the direction of force application, and the yellow arrow representing the movement of the center point of the nucleus, centroid displacement, which is a change in position in the Y-direction. RNAi against *Msp300* reduced centroid displacement relative to the *Luciferase* RNAi control, suggesting impaired force transmission between the cytoskeleton and nucleus. In contrast, RNAi against *Koi* produced no change in nuclear centroid displacement relative to the control. A one-way ANOVA was used to determine statistical significance; 16–28 nuclei were analyzed per genotype. **, *p* ≤ 0.01 **(B)** Inset diagram shows the experimental approach with the black arrowhead representing a micropipette needle, the black arrow indicating the direction of force application, and the yellow double-headed arrow representing the measured change in nuclear deformation. Nuclear strain is measured as the length of the nucleus at the end of the force application minus the length of the nucleus upon the initial force application divided by the length at the end of the force application. RNAi knockdown of the LINC complex components did not alter nuclear strain, indicating that LINC complex disruption did not change nuclear stiffness. A one-way ANOVA was used to determine statistical significance. No statistically significant changes among genotypes were observed; 16–28 nuclei were analyzed per genotype.

Given that depletion of Msp300 caused loss of microtubule caging, we hypothesized that this would lead to an uncoupling of the nucleus from the cytoskeleton like that observed in the LamC ΔK47 and K521Q. To test this hypothesis, we performed microharpooning on muscle fibers expressing either *luciferase*, *Klar,* or *Msp300* RNAi transgenes. The centroid displacement values obtained for muscles depleted for Klar were similar to those of the *luciferase* RNAi control (Figure 7A; Supplementary Videos S5–S7) and consistent with the normal nuclear microtubule caging observed in the Klar depleted muscle. By contrast, depletion of Msp300 caused a significant reduction in centroid displacement relative to that of the control, indicative of a partial loss of nucleo-cytoskeletal coupling (Figure 7A). Motility assays showed that depletion of Msp300 caused reduced larval motility, suggesting a functional significance for coupling between the nucleoskeleton and the cytoskeleton (Supplementary Figure S8). The *Msp300* RNAi transgene is predicted to reduce levels of all known Msp300 isoforms. Given recent findings on isoform-specific functions of Msp300, it is possible that loss of muscle function is due to altered Z disc structure and/or abnormal nuclear positioning (Rey et al., 2021).

**FIGURE 8:**
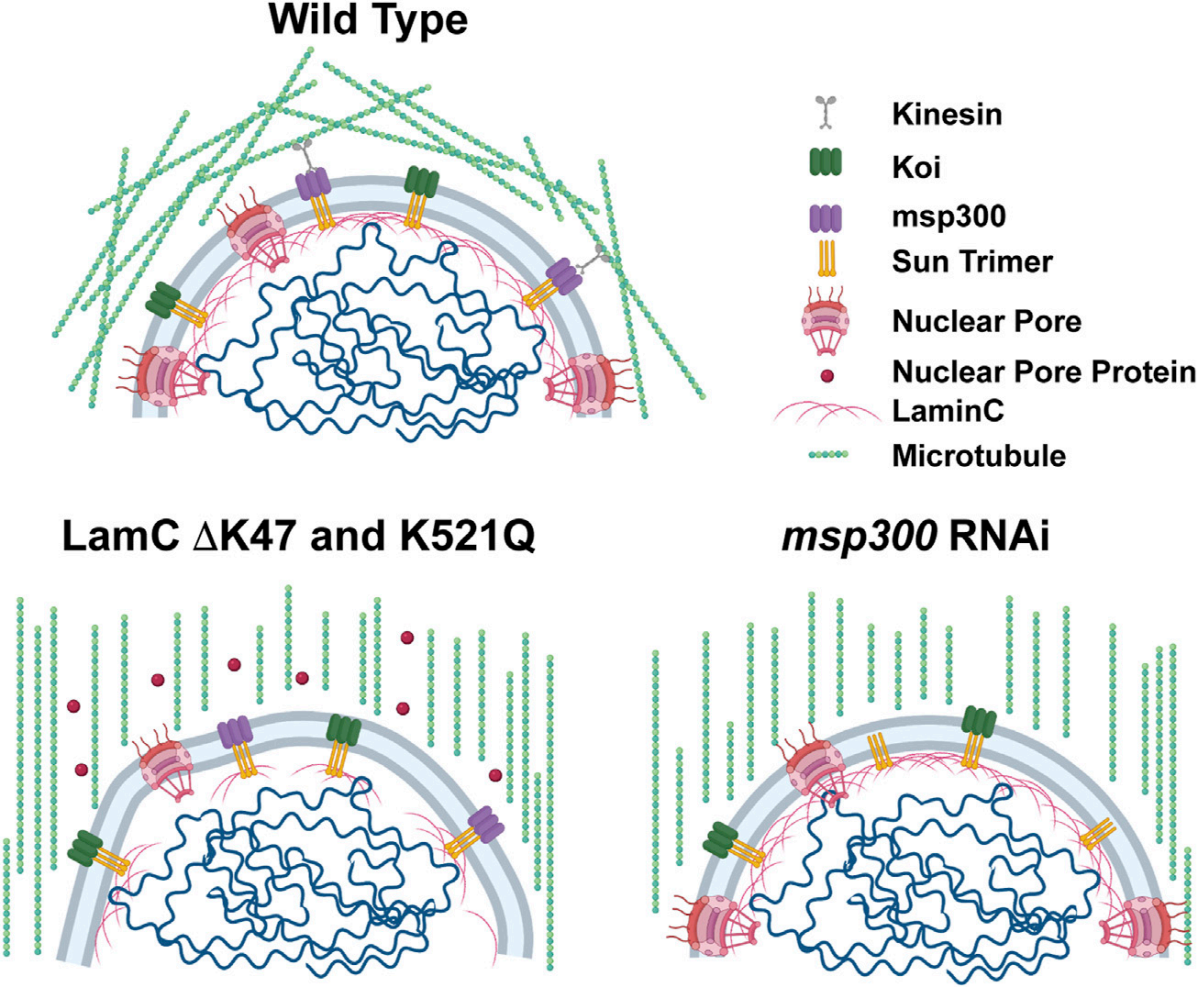
Models show microtubular organization in lamin mutant muscles with impaired nuclear-cytoskeletal connections. Diagram of wild-type muscles in which microtubules form a cage around the nucleus (top). The microtubule cage around the nucleus is reduced in larval body wall muscles expressing either LamC ΔK47 or K521Q (bottom). In these cases, the microtubules run parallel with the long axis of the muscle fiber. In addition, these two mutant lamins cause cytoplasmic mislocalization of nuclear pore proteins (red circles). The microtubule cage around the nucleus is also reduced upon RNAi knockdown of *Msp300*, a KASH-domain LINC complex component, and the microtubules run parallel to the long axis of the muscle fiber. Bio-icons in the model were created with BioRender.com.

Results from the microharpooning assays suggested that Msp300 depletion did not alter nuclear deformation similar to LamC ΔK47 and K521Q, the two mutant lamins that caused uncoupling of the nucleus and cytoskeleton (Figure 7B). Thus, the microharpooning assay functionally distinguished mutants that alter nuclear mechanics from those that do not. Taken together, our experiments indicate that depletion of Msp300 or expression of LamC ΔK47 and K521Q mutants causes impaired nucleo-cytoskeletal force transmission in *Drosophila* body wall muscles by disrupting the nuclear microtubule cage (Figures 5, 6 and 8), establishing an important role of the perinuclear microtubule network organized by Msp300 in transmitting cytoskeletal forces to the myonuclei.

## Discussion

Mutations in *LMNA* cause a plethora of disease phenotypes including skeletal muscular dystrophy and dilated cardiomyopathy (Bonne et al., 1999; Bonne et al., 2000; Hermida-Prieto et al., 2004; Pethig et al., 2005) (Table 1). Individuals with the same *LMNA* mutation can exhibit dramatically different disease presentations and have clinically distinct diagnoses (Muchir et al., 2004; Porcu et al., 2021). This variability can even be observed in closely related family members, strongly implying that phenotypic variability is due to modifier genes. Herein, our goal was to examine muscle defects resulting from specific mutant lamins in a defined genetic background that allows for a direct comparison without the complication of genetic background modifiers. To accomplish this goal, we generated *Drosophila* models of *LMNA*-associated muscular dystrophy. In these models, the mutant lamins display dominant defects, as in human diseases (Worman, 2012). Our studies revealed that specific amino acid changes alter the mechanical functions of myonuclei (Figure 4). Some mutant lamins dramatically affected nuclear deformation, while others impaired nucleo-cytoskeletal coupling. Furthermore, some mutant lamins mislocalized and altered nuclear pore protein localization, while others did not. Collectively, these data demonstrate that the altered phenotypes do not correlate with the domain of lamin affected and that different cellular defects can lead to common premature death in these *Drosophila* models.

Among our observations, we noted that five of the eight amino acid changes in lamin resulted in LamC cytoplasmic aggregation (Figure 2). It is interesting to note that mutant lamins that caused minimal cytoplasmic aggregation altered nuclear shape, a property largely determined by the lamin meshwork (Horn, 2014; Maurer & Lammerding, 2019). Cytoplasmic aggregation of proteins can lead to deleterious consequences for muscle function. Consistent with this idea, increasing rates of autophagy, which reduces cytoplasmic aggregates, suppressed muscle defects caused by mutant lamins in multiple model organisms (Park et al., 2009; Choi et al., 2012; Ramos et al., 2012; Liao et al., 2016; Chiarini et al., 2019; Coombs et al., 2021). In fact*, in silico* studies suggest that lamin aggregation might serve as a predictor of pathogenicity for *LMNA* variants of uncertain significance (Anderson et al., 2021). Seven of the eight amino acid changes in LamC resulted in cytoplasmic aggregation of FG-repeat NUPs (Figure 3). It is unclear if the NUPs are improperly inserted or ineffectively anchored in the nuclear envelope. Regardless, a reduction in functional NUPs is likely to affect nuclear–cytoplasmic transport of biomolecules, causing a myriad of defects. Consistent with the results presented here, FG-repeat NUPs were observed in the cytoplasm of human muscle biopsy tissue from *LMNA* skeletal muscular dystrophy patients, demonstrating clinical relevance (Dialynas et al., 2015).

Herein, we were able to directly assay the functional consequences of mutant lamins on nuclear deformability and nucleo-cytoskeletal coupling *via* a novel application of a microharpooning assay. Three of the mutant lamins (LamC ΔN, L74R, and R205W) showed increased nuclear deformation under force application (Figure 4). In prior studies using a different means of applying nuclear deformation, LamC ΔN was also found to be highly deformable, validating this novel application (Zwerger et al., 2013). Only two mutant lamins (LamC ΔK47 and K521Q) caused a loss of nucleo-cytoskeletal coupling (Figure 4). Surprisingly, these two mutants do not cause overt mislocalization of Koi and Msp300; however, protein–protein interactions could be perturbed by the mutant lamins without gross mislocalization (Supplementary Figure S9). Additionally, these two mutants did not appear to alter nuclear deformation, demonstrating that loss of physical properties needed for coupling is not necessarily required for maintaining nuclear shape upon force application. However, we cannot rule out that these mutations did not increase nuclear deformability, since it is possible that reduced force transmission from the cytoskeleton to the nucleus counteracted this effect.

A particularly noteworthy finding was that nucleo-cytoskeletal uncoupling by specific mutant lamins and depletion of Msp300 were associated with loss of myonuclear microtubule caging (Figures 5, 7 and Figure 8). Our findings are consistent with those of others showing that a network of microtubules around the nucleus in larval body wall muscles is dependent on Msp300 (Volk, 2013). The mechanisms by which microtubules are recruited to the nuclear envelope in differentiated muscle are incompletely understood (Bugnard et al., 2005; Bartolini & Gundersen, 2006; Rogers et al., 2008; Tillery et al., 2018; Becker et al., 2020). In mouse myoblasts undergoing differentiation, centrosomal protein PCM-1 relocalizes to the nuclear envelope by a mechanism that requires Nesprin 1 (Espigat-Georger et al., 2016). Furthermore, the centrosomal protein AKAP450 is required for microtubule nucleation at the nuclear envelope in differentiated myotubes (Gimpel et al., 2017). Consistent with the role of centrosomal proteins in microtubule recruitment, the large scaffold protein AKAP6 links centrosomal protein AKAP9 and nesprin-1α to nucleate microtubules at the nuclear envelope in rat cardiomyocytes (Vergarajauregui et al., 2020). This process is dependent on the induction of muscle-specific isoforms of AKAP6 and nesprin-1α by the transcriptional regulator myogenin (Becker et al., 2020). Similarly, in the *Drosophila* fat body, a network of microtubules around the nucleus is stabilized by several centrosomal proteins and a spectraplakin (Shot) that localize at the nuclear envelope (Wang et al., 2015; Sun et al., 2019; Zheng et al., 2020). It will be of interest to identify the proteins that nucleate microtubules at the nuclear envelope in *Drosophila* larval body wall muscles and investigate how specific mutant lamins alter such interactions.

The functional consequences of the impaired nucleo-cytoskeletal coupling in muscles expressing LamC ΔK47 and K521Q are not known. It is possible that the loss of nuclear microtubule caging reduces larval motility because the non-caged longitudinal microtubules interfere with the actin-myosin contractile apparatus. A dense microtubule network led to increased myocyte stiffness that impaired contractility in failing hearts (Bajpai et al., 2018). In this case, pharmacological treatments leading to the detyrosination of microtubules lowered cytoplasmic viscosity and restored cardiac contractile function. It is also possible that loss of the microtubule nuclear caging in the larval body wall muscle increases nuclear envelope damage, resulting in the loss of muscle function. During muscle contraction, myonuclei move in coordination with the sarcoplasm *via* connections with the cytoskeleton (Lorber et al., 2020). Uncoupling the nucleus from the cytoskeleton can result in asynchronous movements, which generate variable drag forces on myonuclei during muscle contraction (Lorber et al., 2020). Such drag forces might impact normal nuclear mechanosensing and/or lead to nuclear envelope rupture and DNA damage. Consistent with this idea, increased DNA damage has been observed in *LMNA* disease models and human muscle biopsy tissue (Earle et al., 2020). Activation of DNA damage response pathways might signal to block muscle contractions to prevent further DNA damage. In these instances, the microtubule nuclear cage is predicted to serve as a shock absorber, providing protection from mechanical force during muscle contraction. Paradoxically, uncoupling the nucleus from the cytoskeleton *via* disruption of the LINC complex suppresses muscle defects in mouse models of laminopathies (Earle et al., 2020; Chai et al., 2021). In such cases, an altered nuclear lamina might cause nuclei to be especially vulnerable to forces applied from the cytoskeleton; uncoupling the nucleus and cytoskeleton proved beneficial, possibly by reducing forces across the nuclear envelope. Future studies are needed to assess the complex relationship between microtubules and myonuclear integrity.

Only five of the eight mutant lamins studied here showed changes in the physical properties or nucleo-cytoskeletal coupling of myonuclei, as measured by the microharpooning assay, yet all eight caused premature death (Figure 1). The mutant lamins that did not alter nuclear mechanics might play a role in gene expression. Genomes are rich with lamin-associated domains (LADs) in which sections of chromosomes are in close opposition with the lamin meshwork (Mohanta et al., 2021; Wong et al., 2021). In fact, in some cell types, LADs represent up to half of the genome (Briand & Collas, 2020; Mohanta et al., 2021; Rullens & Kind, 2021). In general, LADs are relatively gene poor and lack active transcription. Therefore, the mutant lamins studied here might form an abnormal lamin meshwork that alters contacts with the genome, resulting in altered gene expression.

Collectively, our studies highlight the vast heterogeneity in muscle defects caused by mutant lamins while keeping the genetic background constant. As with other studies of lamins, specific cellular defects do not correlate with alterations in specific protein domains of lamin. Therefore, predictions of how *LMNA* variants of uncertain significance alter lamin function are challenging. Studies such as these will allow for the grouping of mutant lamins that share similar defective properties, which ultimately will guide treatments.

## Data Availability

The original contributions presented in the study are included in the article/Supplementary Material; further inquiries can be directed to the corresponding author.

## References

[B1] Anderson C. L. Langer E. R. Routes T. C. McWilliams S. F. Bereslavskyy I. Kamp T. J. (2021). Most myopathic lamin variants aggregate: A functional genomics approach for assessing variants of uncertain significance. NPJ Genom. Med. 6 (1), 103. 10.1038/s41525-021-00265-x 34862408 PMC8642518

[B122] Arbustini E. Pilotto A. Repetto A. Grasso M. Negri A. Diegoli M. (2002). Autosomal dominant dilated cardiomyopathy with atrioventricular block: a lamin A/C defect-related disease. J. Am. Coll. Cardiol. 39 (6), 981–990. 10.1016/s0735-1097(02)01724-2 11897440

[B2] Astejada M. N. Goto K. Nagano A. Ura S. Noguchi S. Nonaka I. (2007). Emerinopathy and laminopathy clinical, pathological and molecular features of muscular dystrophy with nuclear envelopathy in Japan. Acta Myol. 26 (3), 159–164. 18646565 PMC2949309

[B3] Bajpai G. Schneider C. Wong N. Bredemeyer A. Hulsmans M. Nahrendorf M. (2018). The human heart contains distinct macrophage subsets with divergent origins and functions. Nat. Med. 24 (8), 1234–1245. 10.1038/s41591-018-0059-x 29892064 PMC6082687

[B4] Balakrishnan M. Sisso W. J. Baylies M. K. (2021). Analyzing muscle structure and function throughout the larval instars in live Drosophila. Star. Protoc. 2 (1), 100291. 10.1016/j.xpro.2020.100291 33532738 PMC7821049

[B5] Bartolini F. Gundersen G. G. (2006). Generation of noncentrosomal microtubule arrays. J. Cell Sci. 119 (20), 4155–4163. 10.1242/jcs.03227 17038542

[B6] Becker R. Leone M. Engel F. B. (2020). Microtubule organization in striated muscle cells. Cells 9 (6), E1395. 10.3390/cells9061395 32503326 PMC7349303

[B7] Bobylev A. G. Fadeev R. S. Bobyleva L. G. Kobyakova M. I. Shlyapnikov Y. M. Popov D. V. (2021). Amyloid aggregates of smooth-muscle Titin impair cell adhesion. Int. J. Mol. Sci. 22 (9), 4579. 10.3390/ijms22094579 33925514 PMC8123791

[B8] Bonne G. Di Barletta M. R. Varnous S. Becane H. M. Hammouda E. H. Merlini L. (1999). Mutations in the gene encoding lamin A/C cause autosomal dominant Emery-Dreifuss muscular dystrophy. Nat. Genet. 21 (3), 285–288. 10.1038/6799 10080180

[B9] Bonne G. Mercuri E. Muchir A. Urtizberea A. Becane H. M. Recan D. (2000). Clinical and molecular genetic spectrum of autosomal dominant Emery-Dreifuss muscular dystrophy due to mutations of the lamin A/C gene. Ann. Neurol. 48 (2), 170–180. 10.1002/1531-8249(200008)48:2<170::aid-ana6>3.0.co;2-j 10939567

[B10] Brand A. H. Perrimon N. (1993). Targeted gene expression as a means of altering cell fates and generating dominant phenotypes. Development 118 (2), 401–415. 10.1242/dev.118.2.401 8223268

[B11] Briand N. Collas P. (2020). Lamina-associated domains: Peripheral matters and internal affairs. Genome Biol. 21 (1), 85. 10.1186/s13059-020-02003-5 32241294 PMC7114793

[B12] Brooks D. S. Vishal K. Kawakami J. Bouyain S. Geisbrecht E. R. (2016). Optimization of wrMTrck to monitor Drosophila larval locomotor activity. J. Insect Physiol. 93-94, 11–17. 10.1016/j.jinsphys.2016.07.007 27430166 PMC5722213

[B123] Brown C. A. Lanning R. W. McKinney K. Q. Salvino A. R. Cherniske E. Crowe C. A. (2001). Novel and recurrent mutations in lamin A/C in patients with Emery-Dreifuss muscular dystrophy. Am. J. Med. Genet. 102 (4), 359–367. 10.1002/ajmg.1463 11503164

[B13] Bruusgaard J. C. Liestol K. Gundersen K. (2006). Distribution of myonuclei and microtubules in live muscle fibers of young, middle-aged, and old mice. J. Appl. Physiol. 100 (6), 2024–2030. 10.1152/japplphysiol.00913.2005 16497845

[B124] Budnik V. Zhong Y. Wu C. F. (1990). Morphological plasticity of motor axons in Drosophila mutants with altered excitability. J. Neurosci. 10 (11), 3754–3768. 1700086 10.1523/JNEUROSCI.10-11-03754.1990 PMC6570094

[B14] Bugnard E. Zaal K. J. Ralston E. (2005). Reorganization of microtubule nucleation during muscle differentiation. Cell Motil. Cytoskelet. 60 (1), 1–13. 10.1002/cm.20042 15532031

[B15] Burke B. Stewart C. L. (2006). The laminopathies: The functional architecture of the nucleus and its contribution to disease. Annu. Rev. Genomics Hum. Genet. 7, 369–405. 10.1146/annurev.genom.7.080505.115732 16824021

[B16] Cain N. E. Jahed Z. Schoenhofen A. Valdez V. A. Elkin B. Hao H. (2018). Conserved SUN-KASH interfaces mediate LINC complex-dependent nuclear movement and positioning. Curr. Biol. 28 (19), 3086–3097. e3084. 10.1016/j.cub.2018.08.001 30245107 PMC6219386

[B17] Camozzi D. Capanni C. Cenni V. Mattioli E. Columbaro M. Squarzoni S. (2014). Diverse lamin-dependent mechanisms interact to control chromatin dynamics. Focus on laminopathies. Nucleus 5 (5), 427–440. 10.4161/nucl.36289 25482195 PMC4164485

[B18] Carayon A. Bataille L. Lebreton G. Dubois L. Pelletier A. Carrier Y. (2020). Intrinsic control of muscle attachment sites matching. Elife 9, e57547. 10.7554/eLife.57547 32706334 PMC7431191

[B19] Caygill E. E. Brand A. H. (2016). The GAL4 system: A versatile system for the manipulation and analysis of gene expression. Methods Mol. Biol. 1478, 33–52. 10.1007/978-1-4939-6371-3_2 27730574

[B20] Chai R. J. Werner H. Li P. Y. Lee Y. L. Nyein K. T. Solovei I. (2021). Disrupting the LINC complex by AAV mediated gene transduction prevents progression of Lamin induced cardiomyopathy. Nat. Commun. 12 (1), 4722. 10.1038/s41467-021-24849-4 34354059 PMC8342462

[B21] Chang W. Worman H. J. Gundersen G. G. (2015). Accessorizing and anchoring the LINC complex for multifunctionality. J. Cell Biol. 208 (1), 11–22. 10.1083/jcb.201409047 25559183 PMC4284225

[B22] Chiarini F. Evangelisti C. Cenni V. Fazio A. Paganelli F. Martelli A. M. (2019). The cutting edge: The role of mTOR signaling in laminopathies. Int. J. Mol. Sci. 20 (4), E847. 10.3390/ijms20040847 30781376 PMC6412338

[B23] Chivet M. Marchioretti C. Pirazzini M. Piol D. Scaramuzzino C. Polanco M. J. (2020). Polyglutamine-expanded androgen receptor alteration of skeletal muscle homeostasis and myonuclear aggregation are affected by Sex, Age and muscle metabolism. Cells 9 (2), E325. 10.3390/cells9020325 32019272 PMC7072234

[B24] Cho A. Kato M. Whitwam T. Kim J. H. Montell D. J. (2016). An atypical tropomyosin in Drosophila with intermediate filament-like properties. Cell Rep. 16 (4), 928–938. 10.1016/j.celrep.2016.06.054 27396338 PMC4971881

[B25] Cho S. Irianto J. Discher D. E. (2017). Mechanosensing by the nucleus: From pathways to scaling relationships. J. Cell Biol. 216 (2), 305–315. 10.1083/jcb.201610042 28043971 PMC5294790

[B26] Choi J. C. Muchir A. Wu W. Iwata S. Homma S. Morrow J. P. (2012). Temsirolimus activates autophagy and ameliorates cardiomyopathy caused by lamin A/C gene mutation. Sci. Transl. Med. 4 (144), 144ra102. 144ra102. 10.1126/scitranslmed.3003875 PMC3700376 22837537

[B27] Coombs G. S. Rios-Monterrosa J. L. Lai S. Dai Q. Goll A. C. Ketterer M. R. (2021). Modulation of muscle redox and protein aggregation rescues lethality caused by mutant lamins. Redox Biol. 48, 102196. 10.1016/j.redox.2021.102196 34872044 PMC8646998

[B28] Crisp M. Liu Q. Roux K. Rattner J. B. Shanahan C. Burke B. (2006). Coupling of the nucleus and cytoplasm: Role of the LINC complex. J. Cell Biol. 172 (1), 41–53. 10.1083/jcb.200509124 16380439 PMC2063530

[B29] Dhe-Paganon S. Werner E. D. Chi Y. I. Shoelson S. E. (2002). Structure of the globular tail of nuclear lamin. J. Biol. Chem. 277 (20), 17381–17384. 10.1074/jbc.C200038200 11901143

[B30] Dialynas G. Shrestha O. K. Ponce J. M. Zwerger M. Thiemann D. A. Young G. H. (2015). Myopathic lamin mutations cause reductive stress and activate the nrf2/keap-1 pathway. PLoS Genet. 11 (5), e1005231. 10.1371/journal.pgen.1005231 25996830 PMC4440730

[B31] Dialynas G. Speese S. Budnik V. Geyer P. K. Wallrath L. L. (2010). The role of Drosophila Lamin C in muscle function and gene expression. Development 137 (18), 3067–3077. 10.1242/dev.048231 20702563 PMC2926956

[B32] Dittmer T. A. Misteli T. (2011). The lamin protein family. Genome Biol. 12 (5), 222. 10.1186/gb-2011-12-5-222 21639948 PMC3219962

[B33] Dreger M. Bengtsson L. Schoneberg T. Otto H. Hucho F. (2001). Nuclear envelope proteomics: Novel integral membrane proteins of the inner nuclear membrane. Proc. Natl. Acad. Sci. U. S. A. 98 (21), 11943–11948. 10.1073/pnas.211201898 11593002 PMC59747

[B125] D’Amico A. Haliloglu G. Richard P. Talim B. Maugenre S. Ferreiro A. (2005). Two patients with “Dropped head syndrome” due to mutations in LMNA or SEPN1 genes. Neuromuscul. Disord. 15 (8), 521–524. 10.1016/j.nmd.2005.03.006 15961312

[B34] Earle A. J. Kirby T. J. Fedorchak G. R. Isermann P. Patel J. Iruvanti S. (2020). Mutant lamins cause nuclear envelope rupture and DNA damage in skeletal muscle cells. Nat. Mat. 19 (4), 464–473. 10.1038/s41563-019-0563-5 PMC7102937 31844279

[B35] Eldirany S. A. Lomakin I. B. Ho M. Bunick C. G. (2021). Recent insight into intermediate filament structure. Curr. Opin. Cell Biol. 68, 132–143. 10.1016/j.ceb.2020.10.001 33190098 PMC7925366

[B36] Espigat-Georger A. Dyachuk V. Chemin C. Emorine L. Merdes A. (2016). Nuclear alignment in myotubes requires centrosome proteins recruited by nesprin-1. J. Cell Sci. 129 (22), 4227–4237. 10.1242/jcs.191767 27802164

[B37] Fedorchak G. Lammerding J. (2016). Cell microharpooning to study nucleo-cytoskeletal coupling. Methods Mol. Biol. 1411, 241–254. 10.1007/978-1-4939-3530-7_16 27147047 PMC5966280

[B38] Folker E. S. Ostlund C. Luxton G. W. Worman H. J. Gundersen G. G. (2011). Lamin A variants that cause striated muscle disease are defective in anchoring transmembrane actin-associated nuclear lines for nuclear movement. Proc. Natl. Acad. Sci. U. S. A. 108 (1), 131–136. 10.1073/pnas.1000824108 21173262 PMC3017140

[B39] Fortier T. M. Vasa P. P. Woodard C. T. (2003). Orphan nuclear receptor betaFTZ-F1 is required for muscle-driven morphogenetic events at the prepupal-pupal transition in *Drosophila melanogaster* . Dev. Biol. 257 (1), 153–165. 10.1016/s0012-1606(03)00036-8 12710964

[B40] Furukawa K. Ishida K. Tsunoyama T. A. Toda S. Osoda S. Horigome T. (2009). A-type and B-type lamins initiate layer assembly at distinct areas of the nuclear envelope in living cells. Exp. Cell Res. 315 (7), 1181–1189. 10.1016/j.yexcr.2008.12.024 19210986

[B41] Gimpel P. Lee Y. L. Sobota R. M. Calvi A. Koullourou V. Patel R. (2017). Nesprin-1α-Dependent microtubule nucleation from the nuclear envelope via Akap450 is necessary for nuclear positioning in muscle cells. Curr. Biol. 27 (19), 2999–3009. e2999. 10.1016/j.cub.2017.08.031 28966089 PMC5640514

[B42] Goldman R. D. Shumaker D. K. Erdos M. R. Eriksson M. Goldman A. E. Gordon L. B. (2004). Accumulation of mutant lamin A causes progressive changes in nuclear architecture in Hutchinson-Gilford progeria syndrome. Proc. Natl. Acad. Sci. U. S. A. 101 (24), 8963–8968. 10.1073/pnas.0402943101 15184648 PMC428455

[B43] Gorczyca D. Ashley J. Speese S. Gherbesi N. Thomas U. Gundelfinger E. (2007). Postsynaptic membrane addition depends on the Discs-Large-interacting t-SNARE Gtaxin. J. Neurosci. 27 (5), 1033–1044. 10.1523/JNEUROSCI.3160-06.2007 17267557 PMC4664082

[B44] Hatch E. M. Hetzer M. W. (2016). Nuclear envelope rupture is induced by actin-based nucleus confinement. J. Cell Biol. 215 (1), 27–36. 10.1083/jcb.201603053 27697922 PMC5057282

[B45] Heller S. A. Shih R. Kalra R. Kang P. B. (2020). Emery-Dreifuss muscular dystrophy. Muscle Nerve 61 (4), 436–448. 10.1002/mus.26782 31840275 PMC7154529

[B46] Hermida-Prieto M. Monserrat L. Castro-Beiras A. Laredo R. Soler R. Peteiro J. (2004). Familial dilated cardiomyopathy and isolated left ventricular noncompaction associated with lamin A/C gene mutations. Am. J. Cardiol. 94 (1), 50–54. 10.1016/j.amjcard.2004.03.029 15219508

[B47] Herrmann H. Bar H. Kreplak L. Strelkov S. V. Aebi U. (2007). Intermediate filaments: From cell architecture to nanomechanics. Nat. Rev. Mol. Cell Biol. 8 (7), 562–573. 10.1038/nrm2197 17551517

[B48] Herrmann H. Strelkov S. V. (2011). History and phylogeny of intermediate filaments: Now in insects. BMC Biol. 9, 16. 10.1186/1741-7007-9-16 21356127 PMC3046923

[B49] Hieda M. (2019). Signal transduction across the nuclear envelope: Role of the LINC complex in bidirectional signaling. Cells 8 (2), E124. 10.3390/cells8020124 30720758 PMC6406650

[B50] Hinz B. E. Walker S. G. Xiong A. Gogal R. A. Schnieders M. J. Wallrath L. L. (2021). *In silico* and *in Vivo* analysis of amino acid substitutions that cause laminopathies. Int. J. Mol. Sci. 22 (20), 11226. 10.3390/ijms222011226 34681887 PMC8536974

[B51] Hodzic D. M. Yeater D. B. Bengtsson L. Otto H. Stahl P. D. (2004). Sun2 is a novel mammalian inner nuclear membrane protein. J. Biol. Chem. 279 (24), 25805–25812. 10.1074/jbc.M313157200 15082709

[B52] Horn H. F. (2014). LINC complex proteins in development and disease. Curr. Top. Dev. Biol. 109, 287–321. 10.1016/b978-0-12-397920-9.00004-4 24947240

[B53] Kapinos L. E. Schumacher J. Mucke N. Machaidze G. Burkhard P. Aebi U. (2010). Characterization of the head-to-tail overlap complexes formed by human lamin A, B1 and B2 "half-minilamin" dimers. J. Mol. Biol. 396 (3), 719–731. 10.1016/j.jmb.2009.12.001 20004208

[B54] Kim D. I. Birendra K. C. Roux K. J. (2015). Making the LINC: SUN and KASH protein interactions. Biol. Chem. 396 (4), 295–310. 10.1515/hsz-2014-0267 25720065 PMC4386892

[B55] Kim J. K. Louhghalam A. Lee G. Schafer B. W. Wirtz D. Kim D. H. (2017). Nuclear lamin A/C harnesses the perinuclear apical actin cables to protect nuclear morphology. Nat. Commun. 8 (1), 2123. 10.1038/s41467-017-02217-5 29242553 PMC5730574

[B56] Kittisopikul M. Shimi T. Tatli M. Tran J. R. Zheng Y. Medalia O. (2021). Computational analyses reveal spatial relationships between nuclear pore complexes and specific lamins. J. Cell Biol. 220 (4), e202007082. 10.1083/jcb.202007082 33570570 PMC7883741

[B57] Kracklauer M. P. Banks S. M. Xie X. Wu Y. Fischer J. A. (2007). Drosophila klaroid encodes a SUN domain protein required for Klarsicht localization to the nuclear envelope and nuclear migration in the eye. Fly. (Austin) 1 (2), 75–85. 10.4161/fly.4254 18820457

[B58] Krimm I. Ostlund C. Gilquin B. Couprie J. Hossenlopp P. Mornon J. P. (2002). The Ig-like structure of the C-terminal domain of lamin A/C, mutated in muscular dystrophies, cardiomyopathy, and partial lipodystrophy. Structure 10 (6), 811–823. 10.1016/s0969-2126(02)00777-3 12057196

[B59] Lemke S. B. Schnorrer F. (2018). *In vivo* imaging of muscle-tendon morphogenesis in Drosophila pupae. J. Vis. Exp. 132, 57312. 10.3791/57312 PMC5912364 29443094

[B60] Liao C. Y. Anderson S. S. Chicoine N. H. Mayfield J. R. Academia E. C. Wilson J. A. (2016). Rapamycin reverses metabolic Deficits in lamin A/C-deficient mice. Cell Rep. 17 (10), 2542–2552. 10.1016/j.celrep.2016.10.040 27926859 PMC6594831

[B61] Lilina A. V. Chernyatina A. A. Guzenko D. Strelkov S. V. (2020). Lateral A11 type tetramerization in lamins. J. Struct. Biol. 209 (1), 107404. 10.1016/j.jsb.2019.10.006 31610238

[B62] Lombardi M. L. Jaalouk D. E. Shanahan C. M. Burke B. Roux K. J. Lammerding J. (2011a). The interaction between nesprins and sun proteins at the nuclear envelope is critical for force transmission between the nucleus and cytoskeleton. J. Biol. Chem. 286 (30), 26743–26753. 10.1074/jbc.M111.233700 21652697 PMC3143636

[B63] Lombardi M. L. Zwerger M. Lammerding J. (2011b). Biophysical assays to probe the mechanical properties of the interphase cell nucleus: Substrate strain application and microneedle manipulation. J. Vis. Exp. 55, 3087. 10.3791/3087 PMC3230193 21946671

[B64] Lorber D. Rotkopf R. Volk T. (2020). A minimal constraint device for imaging nuclei in live Drosophila contractile larval muscles reveals novel nuclear mechanical dynamics. Lab. Chip 20 (12), 2100–2112. 10.1039/d0lc00214c 32432302

[B65] Lu J. T. Muchir A. Nagy P. L. Worman H. J. (2011). LMNA cardiomyopathy: Cell biology and genetics meet clinical medicine. Dis. Model. Mech. 4 (5), 562–568. 10.1242/dmm.006346 21810905 PMC3180218

[B66] Maggi L. Carboni N. Bernasconi P. (2016). Skeletal muscle laminopathies: A review of clinical and molecular features. Cells 5 (3), E33. 10.3390/cells5030033 27529282 PMC5040975

[B67] Maire P. Dos Santos M. Madani R. Sakakibara I. Viaut C. Wurmser M. (2020). Myogenesis control by SIX transcriptional complexes. Semin. Cell Dev. Biol. 104, 51–64. 10.1016/j.semcdb.2020.03.003 32247726

[B68] Maniotis A. J. Chen C. S. Ingber D. E. (1997). Demonstration of mechanical connections between integrins, cytoskeletal filaments, and nucleoplasm that stabilize nuclear structure. Proc. Natl. Acad. Sci. U. S. A. 94 (3), 849–854. 10.1073/pnas.94.3.849 9023345 PMC19602

[B69] Maurer M. Lammerding J. (2019). The driving force: Nuclear mechanotransduction in cellular function, fate, and disease. Annu. Rev. Biomed. Eng. 21, 443–468. 10.1146/annurev-bioeng-060418-052139 30916994 PMC6815102

[B70] Meinke P. Mattioli E. Haque F. Antoku S. Columbaro M. Straatman K. R. (2014). Muscular dystrophy-associated SUN1 and SUN2 variants disrupt nuclear-cytoskeletal connections and myonuclear organization. PLoS Genet. 10 (9), e1004605. 10.1371/journal.pgen.1004605 25210889 PMC4161305

[B71] Mohanta T. K. Mishra A. K. Al-Harrasi A. (2021). The 3D genome: From structure to function. Int. J. Mol. Sci. 22 (21), 11585. 10.3390/ijms222111585 34769016 PMC8584255

[B72] Mounkes L. Kozlov S. Burke B. Stewart C. L. (2003). The laminopathies: Nuclear structure meets disease. Curr. Opin. Genet. Dev. 13 (3), 223–230. 10.1016/s0959-437x(03)00058-3 12787783

[B73] Muchir A. Medioni J. Laluc M. Massart C. Arimura T. van der Kooi A. J. (2004). Nuclear envelope alterations in fibroblasts from patients with muscular dystrophy, cardiomyopathy, and partial lipodystrophy carrying lamin A/C gene mutations. Muscle Nerve 30 (4), 444–450. 10.1002/mus.20122 15372542

[B74] Ovalle W. K. (1987). The human muscle-tendon junction. A morphological study during normal growth and at maturity. Anat. Embryol. 176 (3), 281–294. 10.1007/BF00310184 3631532

[B75] Park Y. E. Hayashi Y. K. Bonne G. Arimura T. Noguchi S. Nonaka I. (2009). Autophagic degradation of nuclear components in mammalian cells. Autophagy 5 (6), 795–804. 10.4161/auto.8901 19550147

[B76] Pethig K. Genschel J. Peters T. Wilhelmi M. Flemming P. Lochs H. (2005). LMNA mutations in cardiac transplant recipients. Cardiology 103 (2), 57–62. 10.1159/000082048 15539782

[B77] Porcu M. Corda M. Pasqualucci D. Binaghi G. Sanna N. Matta G. (2021). A very long-term observation of a family with dilated cardiomyopathy and overlapping phenotype from lamin A/C mutation. J. Cardiovasc. Med. 22 (1), 53–58. 10.2459/JCM.0000000000001060 32740430

[B78] Potter C. Hodzic D. (2018). Analysis of high molecular Weight isoforms of nesprin-1 and nesprin-2 with vertical agarose Gel electrophoresis. Methods Mol. Biol. 1840, 25–33. 10.1007/978-1-4939-8691-0_3 30141035

[B79] Potulska-Chromik A. Jedrzejowska M. Gos M. Rosiak E. Kierdaszuk B. Maruszak A. (2021). Pathogenic mutations and putative phenotype-affecting variants in polish myofibrillar Myopathy patients. J. Clin. Med. 10 (5), 914. 10.3390/jcm10050914 33652732 PMC7956316

[B80] Rajasekaran N. S. Connell P. Christians E. S. Yan L. J. Taylor R. P. Orosz A. (2007). Human alpha B-crystallin mutation causes oxido-reductive stress and protein aggregation cardiomyopathy in mice. Cell 130 (3), 427–439. 10.1016/j.cell.2007.06.044 17693254 PMC2962423

[B81] Ramachandran P. Budnik V. (2010). Dissection of Drosophila larval body-wall muscles. Cold Spring Harb. Protoc. 2010 (8), pdb.prot5469. pdb prot5469. 10.1101/pdb.prot5469 20679378

[B82] Ramos F. J. Chen S. C. Garelick M. G. Dai D. F. Liao C. Y. Schreiber K. H. (2012). Rapamycin reverses elevated mTORC1 signaling in lamin A/C-deficient mice, rescues cardiac and skeletal muscle function, and extends survival. Sci. Transl. Med. 4 (144), 144ra103. 10.1126/scitranslmed.3003802 PMC3613228 22837538

[B83] Rankin J. Ellard S. (2006). The laminopathies: A clinical review. Clin. Genet. 70 (4), 261–274. 10.1111/j.1399-0004.2006.00677.x 16965317

[B84] Rey A. Schaeffer L. Durand B. Morel V. (2021). Drosophila nesprin-1 isoforms differentially contribute to muscle function. Cells 10 (11), 3061. 10.3390/cells10113061 34831284 PMC8616381

[B85] Richier B. Inoue Y. Dobramysl U. Friedlander J. Brown N. H. Gallop J. L. (2018). Integrin signaling downregulates filopodia during muscle-tendon attachment. J. Cell Sci. 131 (16), jcs217133. 10.1242/jcs.217133 30054384 PMC6127725

[B86] Riemer D. Stuurman N. Berrios M. Hunter C. Fisher P. A. Weber K. (1995). Expression of Drosophila lamin C is developmentally regulated: Analogies with vertebrate A-type lamins. J. Cell Sci. 108 (10), 3189–3198. 10.1242/jcs.108.10.3189 7593280

[B87] Rogers G. C. Rusan N. M. Peifer M. Rogers S. L. (2008). A multicomponent assembly pathway contributes to the formation of acentrosomal microtubule arrays in interphase Drosophila cells. Mol. Biol. Cell 19 (7), 3163–3178. 10.1091/mbc.E07-10-1069 18463166 PMC2441692

[B88] Rosen J. N. Baylies M. K. (2017). Myofibrils put the squeeze on nuclei. Nat. Cell Biol. 19 (10), 1148–1150. 10.1038/ncb3618 28960202 PMC5933531

[B89] Rullens P. M. J. Kind J. (2021). Attach and stretch: Emerging roles for genome-lamina contacts in shaping the 3D genome. Curr. Opin. Cell Biol. 70, 51–57. 10.1016/j.ceb.2020.11.006 33360765

[B90] Rzepecki R. Gruenbaum Y. (2018). Invertebrate models of lamin diseases. Nucleus 9 (1), 227–234. 10.1080/19491034.2018.1454166 29557730 PMC5973256

[B91] Schindelin J. Arganda-Carreras I. Frise E. Kaynig V. Longair M. Pietzsch T. (2012). Fiji: An open-source platform for biological-image analysis. Nat. Methods 9 (7), 676–682. 10.1038/nmeth.2019 22743772 PMC3855844

[B92] Schulman V. K. Dobi K. C. Baylies M. K. (2015). Morphogenesis of the somatic musculature in *Drosophila melanogaster* . Wiley Interdiscip. Rev. Dev. Biol. 4 (4), 313–334. 10.1002/wdev.180 25758712 PMC4456235

[B126] Shaffer C. D. Wuller J. M. Elgin S. C. (1994). Raising large quantities of Drosophila for biochemical experiments. Methods Cell Biol. 44, 99–108. 10.1016/s0091-679x(08)60908-5 7707979

[B93] Shimi T. Kittisopikul M. Tran J. Goldman A. E. Adam S. A. Zheng Y. (2015). Structural organization of nuclear lamins A, C, B1, and B2 revealed by superresolution microscopy. Mol. Biol. Cell 26 (22), 4075–4086. 10.1091/mbc.E15-07-0461 26310440 PMC4710238

[B94] Shin J. Y. Worman H. J. (2022). Molecular Pathology of laminopathies. Annu. Rev. Pathol. 17, 159–180. 10.1146/annurev-pathol-042220-034240 34672689 PMC8881990

[B127] Song K. Dube M. P. Lim J. Hwang I. Lee I. Kim J. J. (2007). Lamin A/C mutations associated with familial and sporadic cases of dilated cardiomyopathy in Koreans. Exp. Mol. Med. 39 (1), 114–120. 10.1038/emm.2007.13 17334235

[B95] Sosa B. A. Rothballer A. Kutay U. Schwartz T. U. (2012). LINC complexes form by binding of three KASH peptides to domain interfaces of trimeric SUN proteins. Cell 149 (5), 1035–1047. 10.1016/j.cell.2012.03.046 22632968 PMC3383001

[B96] Starr D. A. (2017). Muscle development: Nucleating microtubules at the nuclear envelope. Curr. Biol. 27 (19), R1071–r1073. 10.1016/j.cub.2017.08.030 29017044 PMC6261613

[B97] Stewart C. L. Kozlov S. Fong L. G. Young S. G. (2007). Mouse models of the laminopathies. Exp. Cell Res. 313 (10), 2144–2156. 10.1016/j.yexcr.2007.03.026 17493612 PMC1949387

[B98] Strelkov S. V. Kreplak L. Herrmann H. Aebi U. (2004). Intermediate filament protein structure determination. Methods Cell Biol. 78, 25–43. 10.1016/s0091-679x(04)78002-4 15646614

[B99] Sun T. Song Y. Dai J. Mao D. Ma M. Ni J. Q. (2019). Spectraplakin shot Maintains perinuclear microtubule organization in Drosophila polyploid cells. Dev. Cell 49 (5), 731–747. 10.1016/j.devcel.2019.03.027 31006649

[B100] Sweeney H. L. Hammers D. W. (2018). Motor proteins. Cold Spring Harb. Perspect. Biol. 10 (2), a021931. 10.1101/cshperspect.a021931 29419405 PMC5793755

[B101] Swift J. Ivanovska I. L. Buxboim A. Harada T. Dingal P. C. Pinter J. (2013). Nuclear lamin-A scales with tissue stiffness and enhances matrix-directed differentiation. Science 341 (6149), 1240104. 10.1126/science.1240104 23990565 PMC3976548

[B128] Sylvius N. Bilinska Z. T. Veinot J. P. Fidzianska A. Bolongo P. M. Poon S. (2005). *In vivo* and *in vitro* examination of the functional significances of novel lamin gene mutations in heart failure patients. J. Med. Genet. 42 (8), 639–647. 10.1136/jmg.2004.023283 16061563 PMC1736117

[B102] Tapscott S. J. Davis R. L. Thayer M. J. Cheng P. F. Weintraub H. Lassar A. B. (1988). MyoD1: A nuclear phosphoprotein requiring a Myc homology region to convert fibroblasts to myoblasts. Science 242 (4877), 405–411. 10.1126/science.3175662 3175662

[B103] Technau M. Roth S. (2008). The Drosophila KASH domain proteins Msp-300 and Klarsicht and the SUN domain protein Klaroid have no essential function during oogenesis. Fly. (Austin) 2 (2), 82–91. 10.4161/fly.6288 18820478

[B104] Tillery M. M. L. Blake-Hedges C. Zheng Y. Buchwalter R. A. Megraw T. L. (2018). Centrosomal and non-centrosomal microtubule-organizing centers (MTOCs) in *Drosophila melanogaster* . Cells 7 (9), E121. 10.3390/cells7090121 30154378 PMC6162459

[B105] Turgay Y. Medalia O. (2017). The structure of lamin filaments in somatic cells as revealed by cryo-electron tomography. Nucleus 8 (5), 475–481. 10.1080/19491034.2017.1337622 28635493 PMC5703231

[B106] Vergarajauregui S. Becker R. Steffen U. Sharkova M. Esser T. Petzold J. (2020). AKAP6 orchestrates the nuclear envelope microtubule-organizing center by linking golgi and nucleus via AKAP9. Elife 9, e61669. 10.7554/eLife.61669 33295871 PMC7725499

[B107] Volk T. (2013). Positioning nuclei within the cytoplasm of striated muscle fiber: Cooperation between microtubules and KASH proteins. Nucleus 4 (1), 18–22. 10.4161/nucl.23086 23211643 PMC3585022

[B129] Walter M. C. Witt T. N. Weigel B. S. Reilich P. Richard P. Pongratz D. (2005). Deletion of the LMNA initiator codon leading to a neurogenic variant of autosomal dominant Emery-Dreifuss muscular dystrophy. Neuromuscul. Disord. 15 (1), 40–44. 10.1016/j.nmd.2004.09.007 15639119

[B108] Wang N. Tytell J. D. Ingber D. E. (2009). Mechanotransduction at a distance: Mechanically coupling the extracellular matrix with the nucleus. Nat. Rev. Mol. Cell Biol. 10 (1), 75–82. 10.1038/nrm2594 19197334

[B109] Wang S. Reuveny A. Volk T. (2015). Nesprin provides elastic properties to muscle nuclei by cooperating with spectraplakin and EB1. J. Cell Biol. 209 (4), 529–538. 10.1083/jcb.201408098 26008743 PMC4442817

[B110] Wang W. Shi Z. Jiao S. Chen C. Wang H. Liu G. (2012). Structural insights into SUN-KASH complexes across the nuclear envelope. Cell Res. 22 (10), 1440–1452. 10.1038/cr.2012.126 22945352 PMC3463262

[B111] Wilson K. L. Foisner R. (2010). Lamin-binding proteins. Cold Spring Harb. Perspect. Biol. 2 (4), a000554. 10.1101/cshperspect.a000554 20452940 PMC2845209

[B130] Wiltshire K. M. Hegele R. A. Innes A. M. Brownell A. K. (2013). Homozygous lamin A/C familial lipodystrophy R482Q mutation in autosomal recessive Emery Dreifuss muscular dystrophy. Neuromuscul. Disord. 23 (3), 265–268. 10.1016/j.nmd.2012.11.011 23313286

[B112] Wong X. Hoskins V. E. Melendez-Perez A. J. Harr J. C. Gordon M. Reddy K. L. (2021). Lamin C is required to establish genome organization after mitosis. Genome Biol. 22 (1), 305. 10.1186/s13059-021-02516-7 34775987 PMC8591896

[B113] Wong X. Melendez-Perez A. J. Reddy K. L. (2022). The nuclear lamina. Cold Spring Harb. Perspect. Biol. 14 (2), a040113. 10.1101/cshperspect.a040113 34400553 PMC8805651

[B114] Worman H. J. Bonne G. (2007). Laminopathies": A wide spectrum of human diseases. Exp. Cell Res. 313 (10), 2121–2133. 10.1016/j.yexcr.2007.03.028 17467691 PMC2964355

[B115] Worman H. J. (2012). Nuclear lamins and laminopathies. J. Pathol. 226 (2), 316–325. 10.1002/path.2999 21953297 PMC6673656

[B116] Xie X. Fischer J. A. (2008). On the roles of the Drosophila KASH domain proteins Msp-300 and Klarsicht. Fly. (Austin) 2 (2), 74–81. 10.4161/fly.6108 18820482

[B117] Yu J. Starr D. A. Wu X. Parkhurst S. M. Zhuang Y. Xu T. (2006). The KASH domain protein MSP-300 plays an essential role in nuclear anchoring during Drosophila oogenesis. Dev. Biol. 289 (2), 336–345. 10.1016/j.ydbio.2005.10.027 16337624

[B118] Zhang Q. Bethmann C. Worth N. F. Davies J. D. Wasner C. Feuer A. (2007). Nesprin-1 and -2 are involved in the pathogenesis of Emery Dreifuss muscular dystrophy and are critical for nuclear envelope integrity. Hum. Mol. Genet. 16 (23), 2816–2833. 10.1093/hmg/ddm238 17761684

[B119] Zhang Q. Skepper J. N. Yang F. Davies J. D. Hegyi L. Roberts R. G. (2001). Nesprins: A novel family of spectrin-repeat-containing proteins that localize to the nuclear membrane in multiple tissues. J. Cell Sci. 114 (24), 4485–4498. 10.1242/jcs.114.24.4485 11792814

[B120] Zheng Y. Buchwalter R. A. Zheng C. Wight E. M. Chen J. V. Megraw T. L. (2020). A perinuclear microtubule-organizing centre controls nuclear positioning and basement membrane secretion. Nat. Cell Biol. 22 (3), 297–309. 10.1038/s41556-020-0470-7 32066907 PMC7161059

[B121] Zwerger M. Jaalouk D. E. Lombardi M. L. Isermann P. Mauermann M. Dialynas G. (2013). Myopathic lamin mutations impair nuclear stability in cells and tissue and disrupt nucleo-cytoskeletal coupling. Hum. Mol. Genet. 22 (12), 2335–2349. 10.1093/hmg/ddt079 23427149 PMC3658163

