# Supplementary material for: Effects of mutant lamins on nucleo-cytoskeletal coupling in Drosophila models of LMNA muscular dystrophy
Source: Front Cell Dev Biol. 2022 Aug 31;10:934586. doi: 10.3389/fcell.2022.934586 (PMC9471154; doi:10.3389/fcell.2022.934586)
Supplement: Supplementary file 3 [file DataSheet1.pdf]

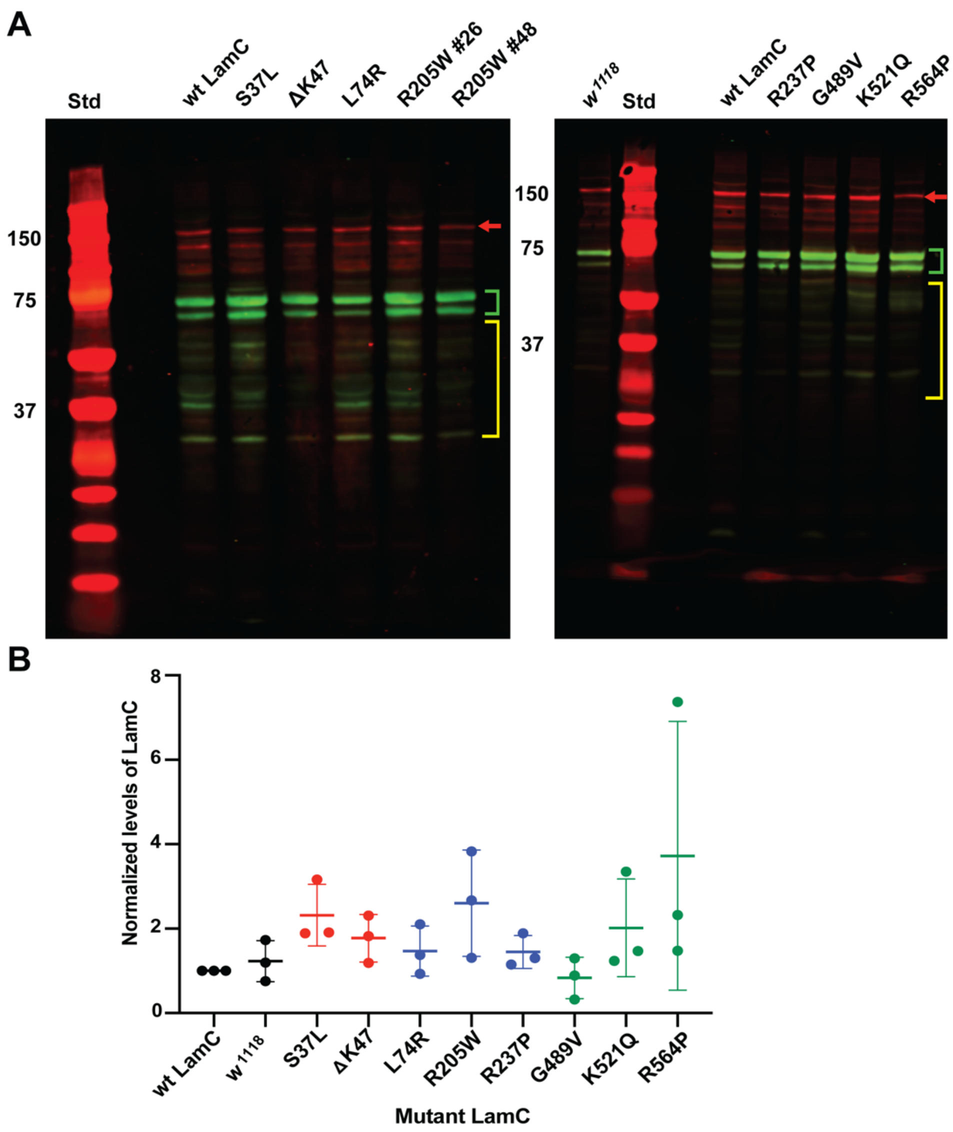

Supplemental Figure 1. Mutant LamC proteins are expressed at similar levels to that of endogenous LamC. (A) Western analysis was performed on protein extracts generated from larval body wall muscles. Membranes were stained with antibodies to LamC (green fluorescence and green bracket) and a uniformly expressed control protein CP190 (red staining and red arrow). Yellow brackets indicate non-specific staining that is also seen in all lanes. (**B**) Ratios of LamC to CP190 were determined for each transgene and were normalized to that of wild-type LamC. A one-way ANOVA was used to determine statistical significance. No statistical differences were determined among the genotypes, indicating that the muscle defects were caused by the mutant lamins and not general overexpression of lamin.

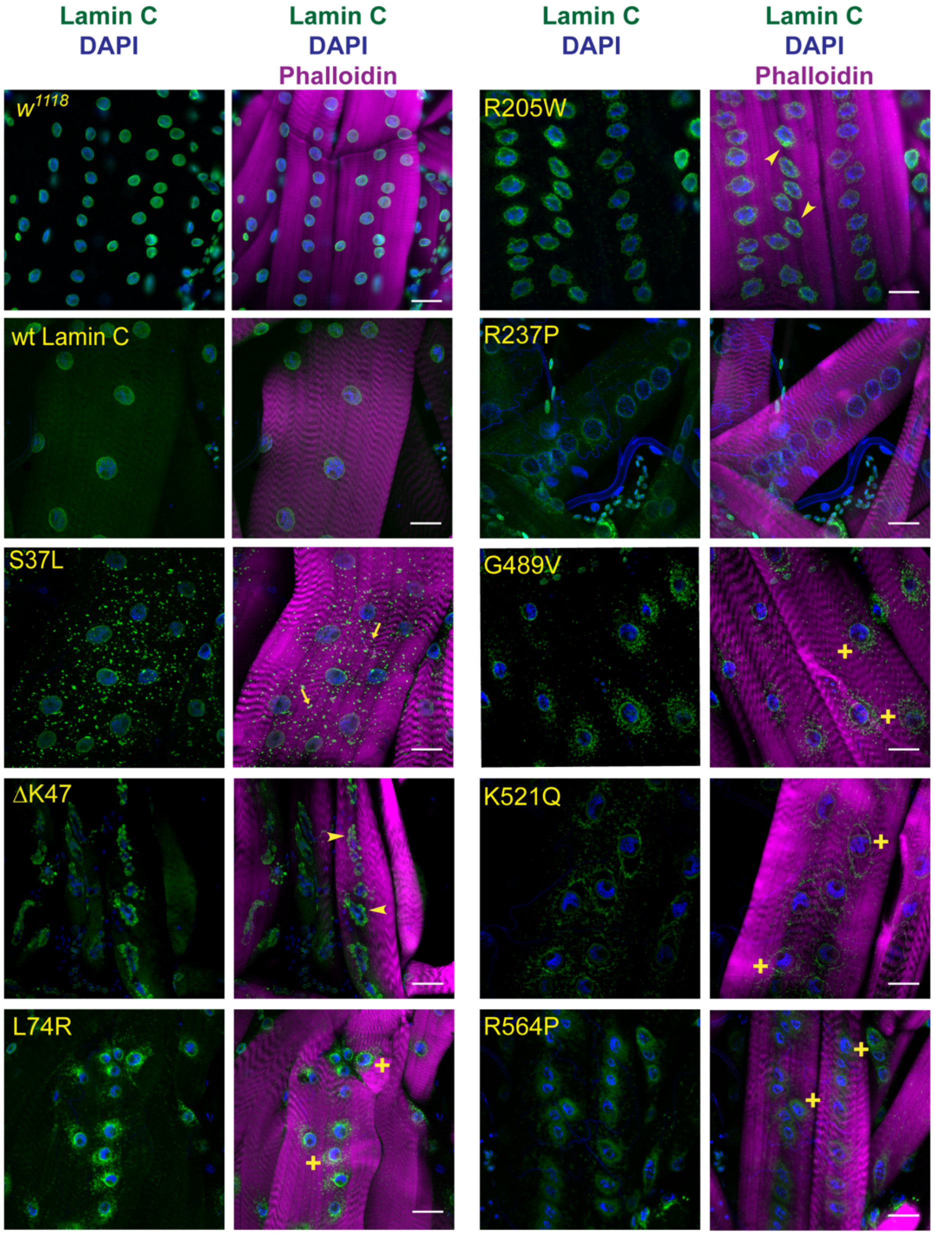

Supplemental Figure 2: Mutant lamins mislocalize in muscle. Either wild-type or mutant *LamC* was expressed in larval body wall muscle using the *C57* Gal4 driver. The muscles were dissected and stained with antibodies to LamC (green), phalloidin (magenta), and DAPI (blue). Arrows denote cytoplasmic aggregation, arrowheads denote nuclear aggregation, and plus signs denote perinuclear aggregation. Alterations in LamC localization, nuclear shape, and nuclear migration were not observed upon expression of wild-type LamC compared to the host injection stock (w^{1118}). In contrast, alterations in nuclear shape were observed upon expression of LamC ΔK47 and R205W, which produced oblong nuclei with nuclear envelope lobulations. In addition, alterations in nuclear migration were observed for LamC R237P, in which the nuclei formed chains with minimal spacing between nuclei. Scale bar: 30 μm.

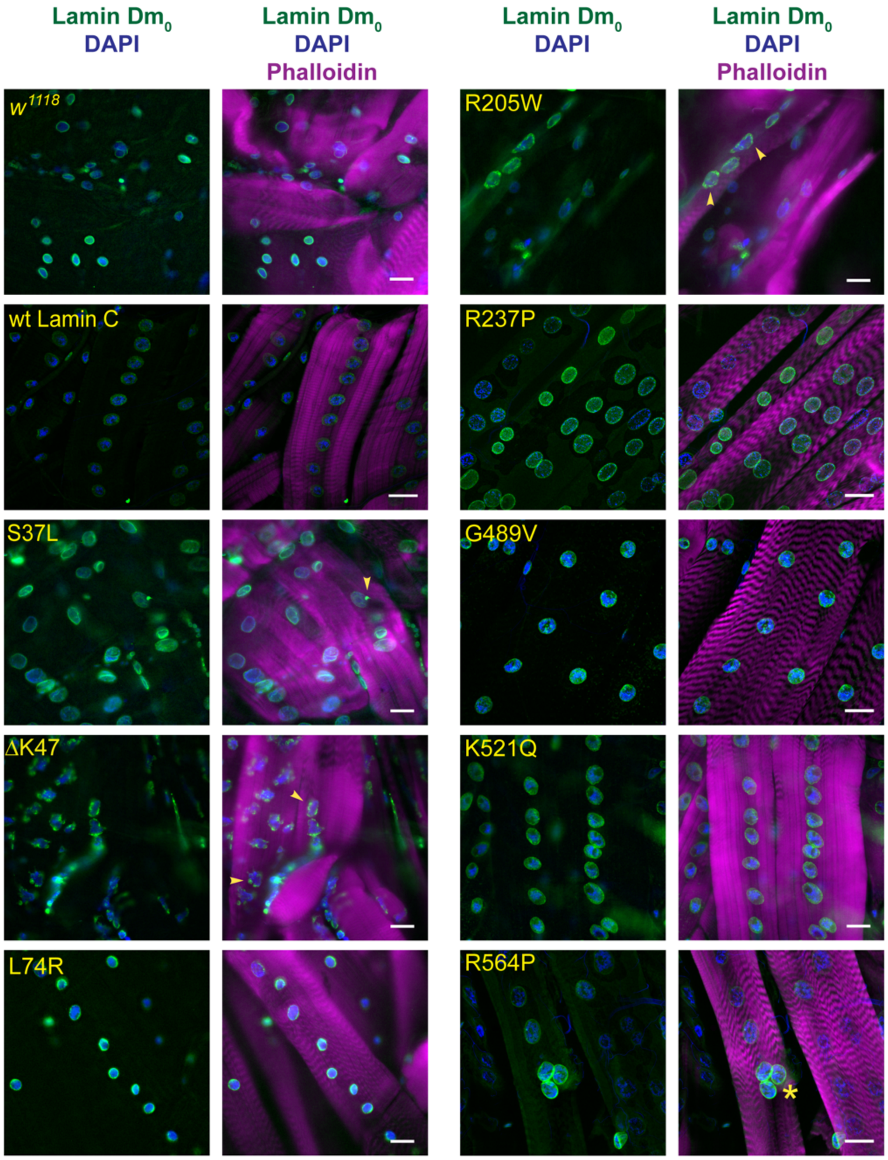

Supplemental Figure 3: Specific mutant versions of LamC cause mislocalization of lamin Dm$_0$. Either wild-type or mutant *LamC* was expressed in larval body wall muscle using the *C57* Gal4 driver. The muscles were dissected and stained with antibodies to lamin Dm$_0$ (green), phalloidin (magenta), and DAPI (blue). Arrowheads denote nuclear aggregation and asterisks denote nuclear clustering. Lamin Dm$_0$ mislocalization was not observed in muscles of the host stock (w^{1118}) and muscles expressing wild-type *LamC*. Nuclear aggregation of lamin Dm$_0$ was observed in muscles expressing LamC S37L, ΔK47, and R205W. In addition, nuclear clustering was observed for R564P. Scale bar: 30 mm.

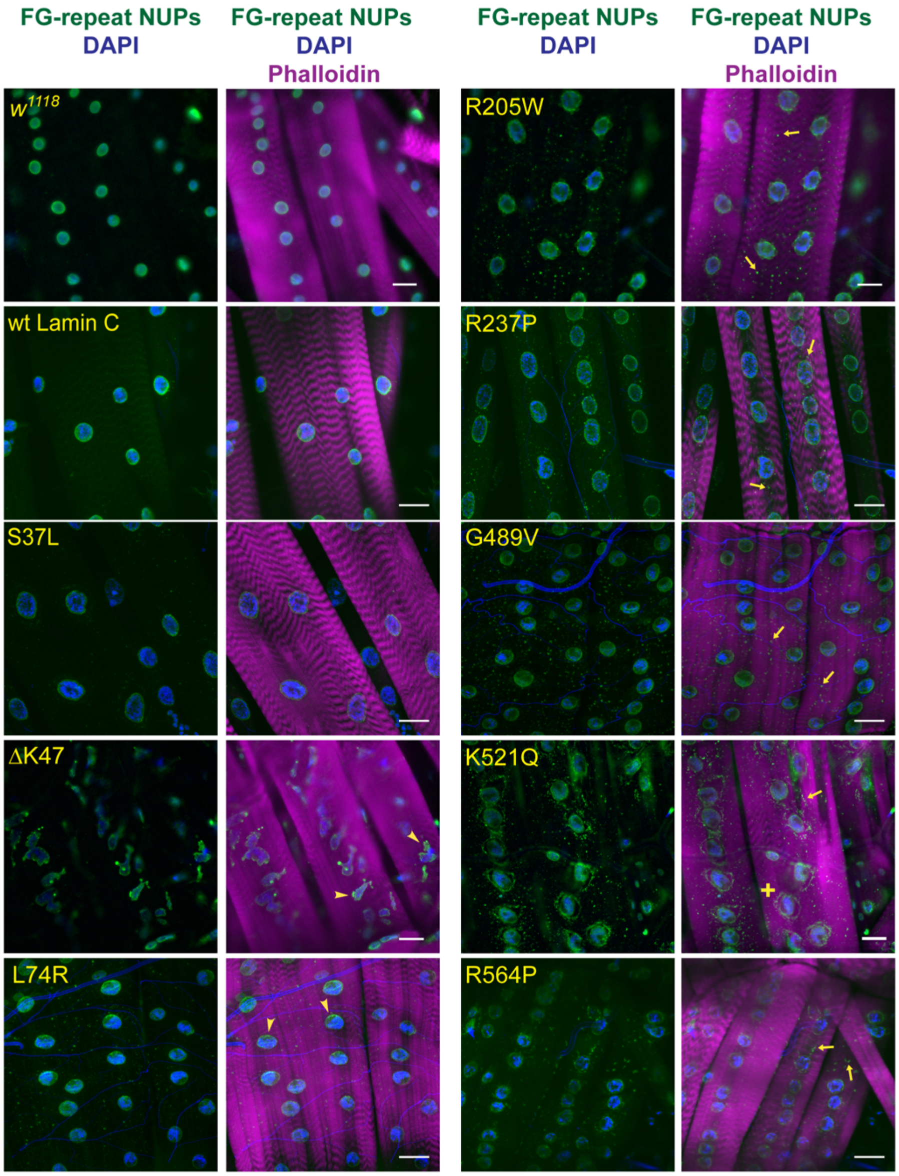

Supplemental Figure 4: Specific mutant versions of LamC alter the localization of FG-containing nuclear pore proteins. Body wall muscles from larvae expressing either wild type or mutant *LamC* were stained with antibodies to LamC (orange) or FG-repeat containing nuclear pore proteins (NUPs) (green), phalloidin (magenta), and DAPI (blue). Staining with antibodies that recognize FG-repeat containing NUPs showed that specific mutant lamins (R237P, R205W, G489V, K521Q, and R564P) caused cytoplasmic aggregations of NUPs (yellow arrows). Other mutant versions of LamC (ΔK47 and L74R) caused nuclear aggregation of FG-repeat containing NUPs (yellow arrowheads). K521Q also showed perinuclear aggregation of NUPs (plus sign). Scale Bar: 30 μm.

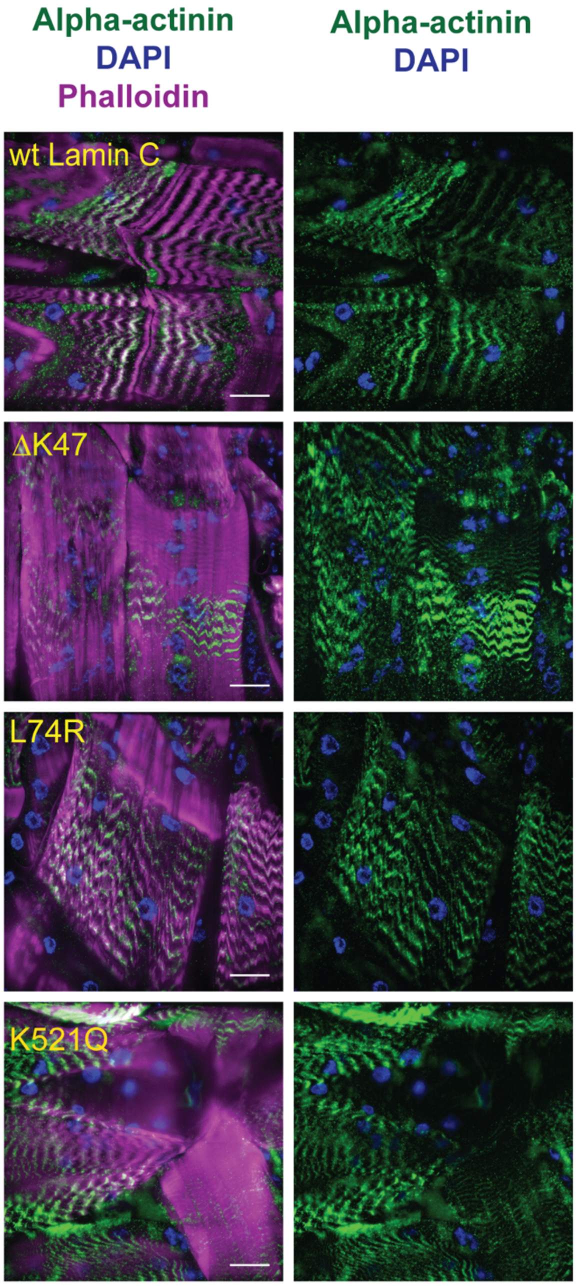

Supplemental Figure 5: Mutant LamC does not alter the localization of alpha-actinin in larval body wall muscles. Larvae expressing either wild-type or mutant LamC were dissected and stained with antibodies to alpha-actinin (green), DAPI (blue) and phalloidin (magenta). In all cases examined, alpha-actinin was observed to exhibit the herringbone pattern consistent with proper localization to Z-bands.

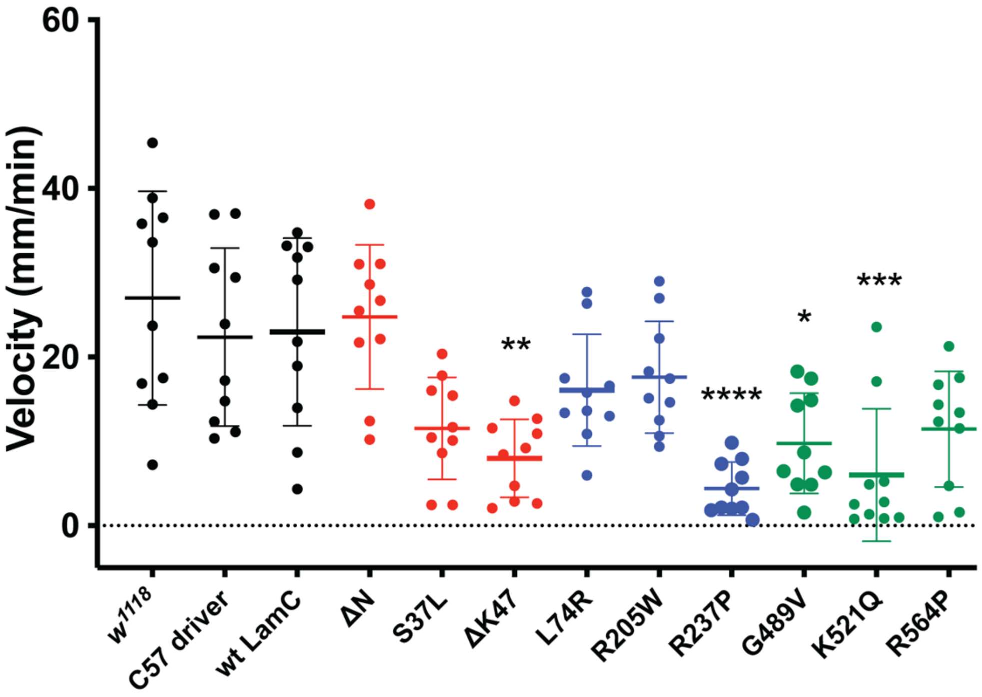

Supplemental Figure 6: Specific mutant versions of LamC alter larval motility. The average velocity was calculated and plotted for ten larvae of each genotype studied. A one-way ANOVA was used to determine significance differences among genotypes. *, $p \le 0.05$; **, $p \le 0.01$; ***, ≤ 0.001; ****, $p \le 0.0001$

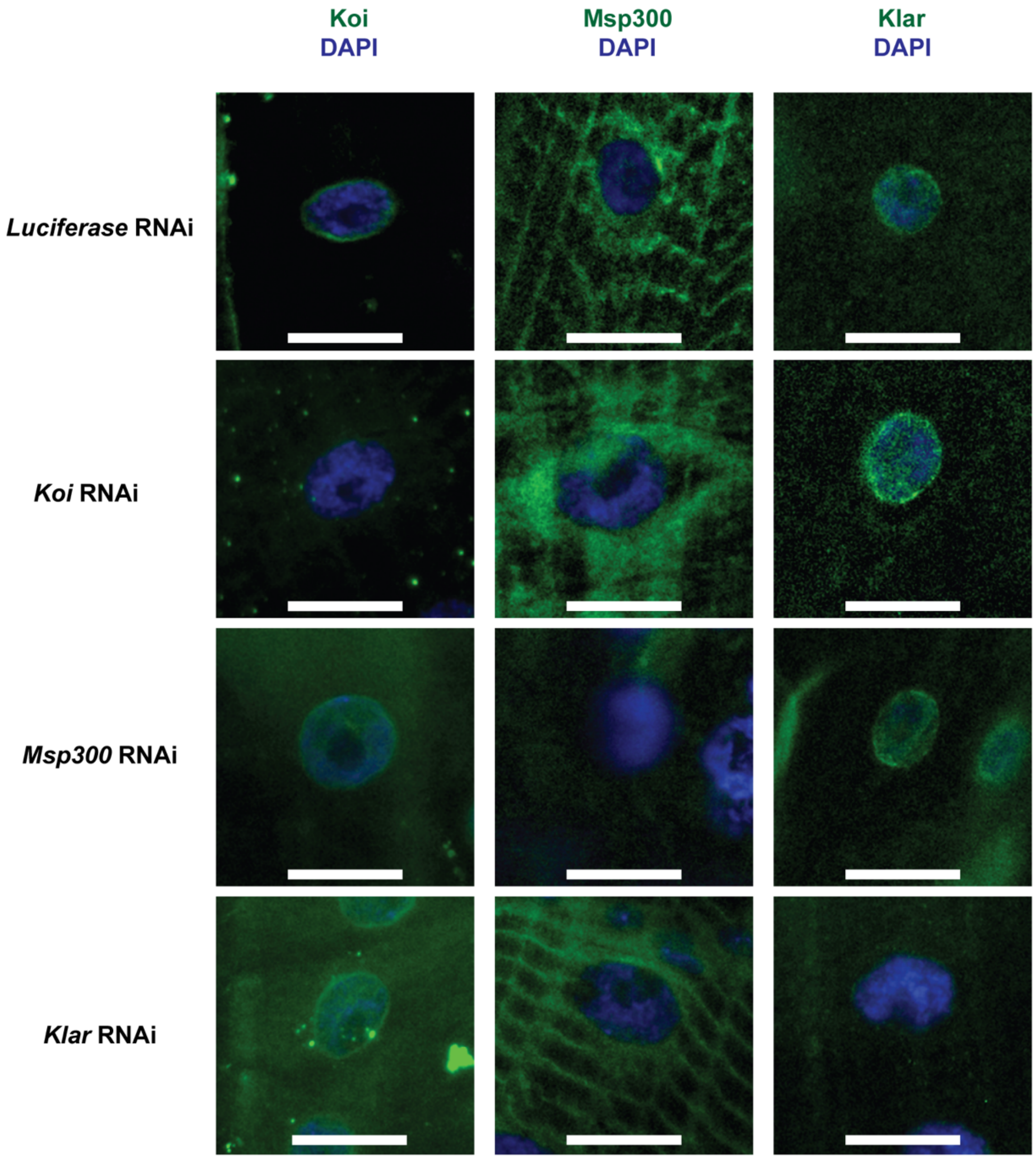

Supplemental Figure 7: Expression of RNAi against LINC complex components provides effective protein knock-down in muscle. Larval body wall muscles expressing transgenes encoding RNAi against LINC complex components were stained with antibodies to the LINC complex proteins (green) and DAPI (blue). Expression of an RNAi against a non-specific control (*Luciferase*) did not disrupt localization of Koi, Msp300, and Klar. Expression of an RNAi against *Koi* caused the anticipated loss of Koi from the nuclear envelope and no change in the localization of Msp300 and Klar. Expression of an RNAi against *Msp300* caused the anticipated loss of Msp300 from the nuclear envelope but did not alter Klar localization. Expression of an RNAi against *Klar* showed the anticipated loss of Klar from the nuclear envelope with no change in the localization of Msp300 and Koi. Scale bar represents 30 mm.

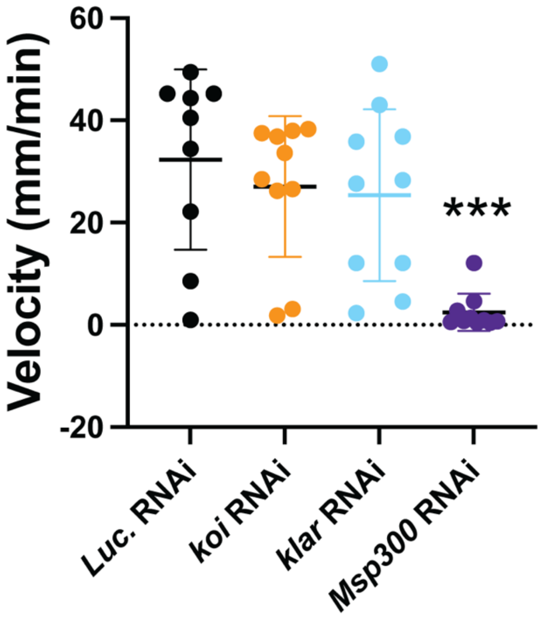

Supplemental Figure 8: Muscle-specific depletion of Msp300 causes reduced larval motility. The average velocity was calculated and plotted for ten larvae of each genotype studied. A one-way ANOVA was used to determine significance differences among genotypes. ***, ≤ 0.001

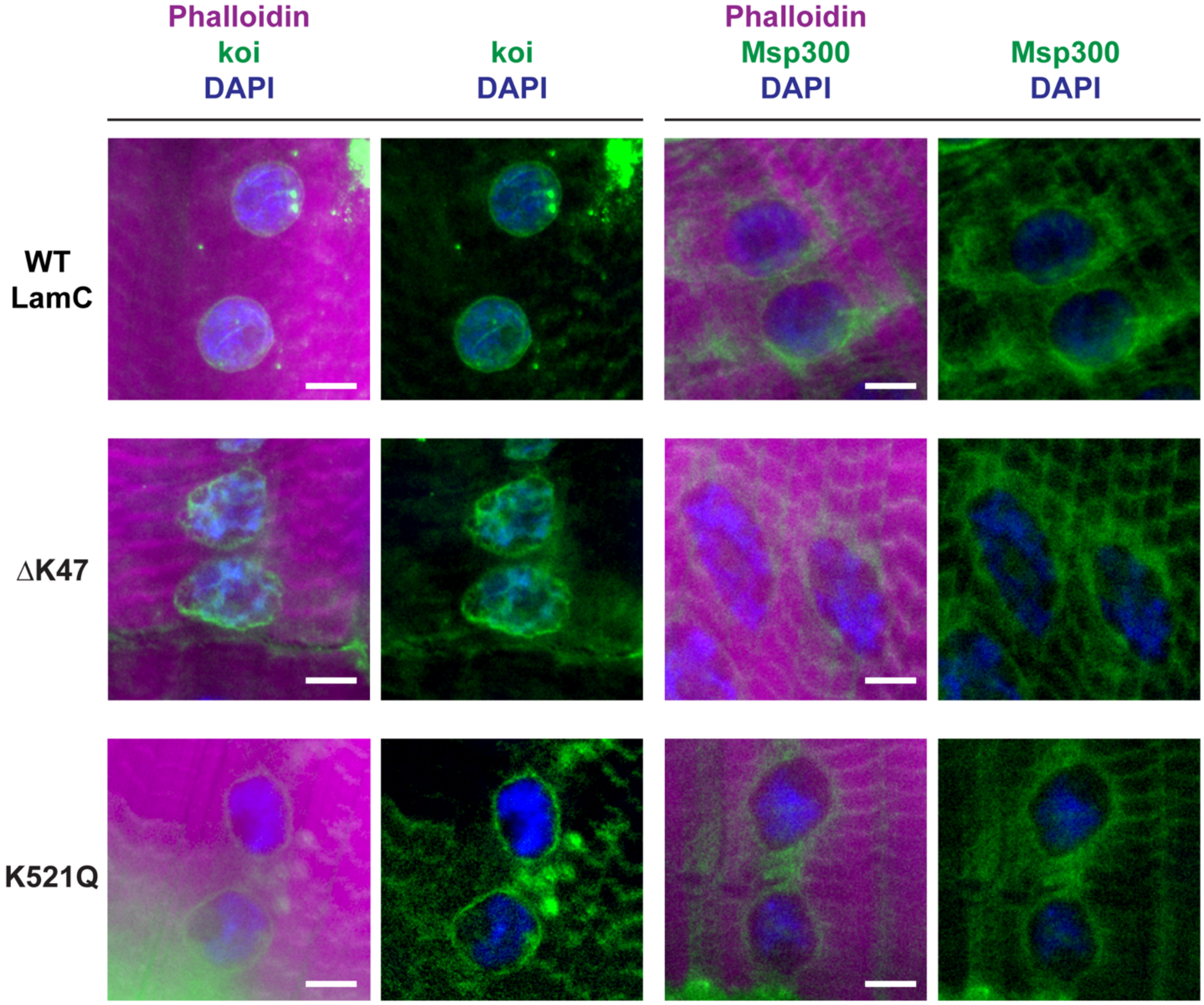

Supplemental Figure 9. Nuclear envelope localization of koi and Msp300 is maintained upon expression of LamC ΔK47 and K521Q. Larval body wall muscles expressing either wild-type LamC, ΔK47 or K521Q were stained with phalloidin (magenta), DAPI (blue) and antibodies to koi or Msp300 (green). The localization of koi and Msp300 was not overtly perturbed in these genetic backgrounds**.** Scale bar indicates 10 μm.

Supplemental Video 1. Microharpooning of larval body wall muscles expressing wild-type LamC. The microharpoon (visualized on the right) was inserted into the cytoskeleton ~10 to15 μm from the edge of the nucleus and pulled 30 μm in the direction away from the nucleus at a rate of two μm/s using custom MATLAB software to control the motorized micromanipulator (Eppendorf InjectMan NI2). Pull direction was along the long axis of the myofiber and away from the nucleus. Images were acquired at 32× magnification (20× objective with 1.6× Optivar) every five seconds.

Supplemental Video 2. Microharpooning of larval body wall muscles expressing LamC L74R. The microharpoon was inserted into the cytoskeleton ~10 to15 μm from the edge of the nucleus (visualized on the right) and pulled 30 μm in the direction away from the nucleus at a rate of two μm/s using custom MATLAB software to control the motorized micromanipulator (Eppendorf InjectMan NI2). Pull direction was along the long axis of the myofiber and away from the nucleus. Images were acquired at 32× magnification (20× objective with 1.6× Optivar) every five seconds. Note the increased nuclear deformation relative to the control in video 1.

Supplemental Video 3. Microhaprooning of larval body wall muscles expressing LamC K521Q. The microharpoon was inserted into the cytoskeleton ~10 to15 μm from the edge of the nucleus (based on crosshairs in the objective) and pulled 30 μm in the direction away from the nucleus at a rate of two μm/s using custom MATLAB software to control the motorized micromanipulator (Eppendorf InjectMan NI2). Pull direction was along the long axis of the myofiber and away from the nucleus. Images were acquired at 32× magnification (20× objective with 1.6× Optivar) every five seconds. Note the lack of centroid displacement compared to the control in video 1.

Supplemental Video 4. Microharpooning of larval body wall muscles expressing LamC R564P. The microharpoon was inserted into the cytoskeleton ~10 to15 μm from the edge of the nucleus (based on crosshairs in the objective) and pulled 30 μm in the direction away from the nucleus at a rate of two μm/s using custom MATLAB software to control the motorized micromanipulator (Eppendorf InjectMan NI2). Pull direction was along the long axis of the myofiber and away from the nucleus. Images were acquired at 32× magnification (20× objective with 1.6× Optivar) every five seconds. Note the similarities in nuclear deformation and centroid displacement to that of the control in video 1.

Supplemental Video 5. Microharpooning of larval body wall muscles expressing an RNAi against *Luciferase* as a control. The microharpoon was inserted into the cytoskeleton ~10 to15 μm from the edge of the nucleus (based on crosshairs in the objective) and pulled 30 μm in the direction away from the nucleus at a rate of two μm/s using custom MATLAB software to control the motorized micromanipulator (Eppendorf InjectMan NI2). Pull direction was along the long axis of the myofiber and away from the nucleus. Images were acquired at 32× magnification (20× objective with 1.6× Optivar) every five seconds.

Supplemental Video 6. Microharpooning of larval body wall muscles depleted of Koi. The microharpoon was inserted into the cytoskeleton ~10 to15 μm from the edge of the nucleus (based on crosshairs in the objective) and pulled 30 μm in the direction away from the nucleus at a rate of two μm/s using custom MATLAB software to control the motorized micromanipulator (Eppendorf InjectMan NI2). Pull direction was along the long axis of the myofiber and away from the nucleus. Images were acquired at 32× magnification (20× objective with 1.6× Optivar) every five seconds. Note that the nuclear deformation and centroid displacement is similar to that of the control in video 5.

Supplemental Video 7. Microharpooning of larval body wall muscles depleted of Msp300. The microharpoon was inserted into the cytoskeleton ~10 to15 μm from the edge of the nucleus (based on crosshairs in the objective) and pulled 30 μm in the direction away from the nucleus at a rate of two μm/s using custom MATLAB software to control the motorized micromanipulator (Eppendorf InjectMan NI2). Pull direction was along the long axis of the myofiber and away from the nucleus. Images were acquired at 32× magnification (20× objective with 1.6× Optivar) every five seconds. Note the lack of centroid displacement compared to that of the control in video 5.
